# High predictability of direct competition between marine diatoms under different temperatures and nutrient states

**DOI:** 10.1002/ece3.6453

**Published:** 2020-06-03

**Authors:** Philipp Siegel, Kirralee G. Baker, Etienne Low‐Décarie, Richard J. Geider

**Affiliations:** ^1^ School of Life Sciences University of Essex Colchester Campus Colchester UK; ^2^ Canadian Space Agency Saint‐Hubert QC Canada

**Keywords:** competition, diatoms, phytoplankton, thermal performance

## Abstract

The distribution of marine phytoplankton will shift alongside changes in marine environments, leading to altered species frequencies and community composition. An understanding of the response of mixed populations to abiotic changes is required to adequately predict how environmental change may affect the future composition of phytoplankton communities. This study investigated the growth and competitive ability of two marine diatoms, *Phaeodactylum tricornutum* and *Thalassiosira pseudonana*, along a temperature gradient (9–35°C) spanning the thermal niches of both species under both high‐nitrogen nutrient‐replete and low‐nitrogen nutrient‐limited conditions. Across this temperature gradient, the competitive outcome under both nutrient conditions at any assay temperature, and the critical temperature at which competitive advantage shifted from one species to the other, was well predicted by the temperature dependencies of the growth rates of the two species measured in monocultures. The temperature at which the competitive advantage switched from *P. tricornutum* to *T. pseudonana* increased from 18.8°C under replete conditions to 25.3°C under nutrient‐limited conditions. Thus, *P. tricornutum* was a better competitor over a wider temperature range in a low N environment. Being able to determine the competitive outcomes from physiological responses of single species to environmental changes has the potential to significantly improve the predictive power of phytoplankton spatial distribution and community composition models.

## Graphical abstract

An understanding of the response of mixed populations to abiotic changes is required in order to adequately predict the future composition of phytoplankton communities. This study investigated the growth and competitive ability of two marine diatoms, *Phaeodactylum tricornutum* and *Thalassiosira pseudonana*, along a temperature gradient spanning the thermal niches of both species (from 9°C to 35°C) under high‐nitrogen and low‐nitrogen conditions. Competitive outcomes between the two diatoms were highly predictable based on their monoculture growth performances, indicating that their isolated performance can be used to infer competitive ability and resulting community structure.

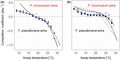

## 1 INTRODUCTION

Competition plays an important role in shaping biological communities in terrestrial and aquatic ecosystems (Tilman, 1987). Interspecific competition within communities occurs when two or more species possess the same or similar resource requirements (Clements & Shelford, 1939). For phytoplankton, the most important resources are macronutrients (N, P, Si), micronutrients (trace elements), organic nutrients (vitamins), CO_2_, and light (Riebesell, 2004; Tilman, Kilham, & Kilham, 1982). If demand for a resource by the organisms within an ecosystem is high, the abundance or concentration of the resource will decline. When the concentration of a resource becomes too low, it can fall below the minimum requirement of a species to support its temperature and light‐dependent maximum growth rate (*µ*_max_) and, therefore, become limiting (Andersen, 2005).

In equilibrium communities, the minimum resource concentration that supports net population growth, and for which uptake rates by the population and supply rates by the environment are in balance, is called R* (Tilman, 1981). Assuming that all species compete for the same limiting resource and ignoring the effects of interference or apparent competition, the species with the lowest R* should outcompete all other species and dominate a community (resource ratio theory or R*‐theory; Tilman et al., 1982). Theoretically, the number of species that can stably coexist in a system is therefore equal to the number of limiting resources, if different species are limited by different resources. The fact that most communities of primary producers usually display higher species diversity than the number of limiting resources (Cloern & Dufford, 2005; Sommer, 1984) has been termed the “Paradox of the plankton” (Hutchinson, 1961). The resolution to the paradox likely lies in the fact that natural communities are often not in equilibrium or that the ability of a species to exploit resources is not the only factor that regulates a community's diversity and the outcome of competition. Apart from limiting nutrients, species diversity is governed by other major regulating forces which can be biotic (e.g., differential grazing pressure or various forms of symbiosis such as mutualism or parasitism) or abiotic (e.g., temperature, pH, salinity) (Begon, Townsend, & Harper, 2006; Cloern & Dufford, 2005).

Whether the effect of environmental change on the outcome of competition can be predicted from the performance of isolated species and whether these predictions align with classical competition models or single species performance is unclear because experiments that examine direct species interactions along environmental gradients are scarce (Kordas, Harley, & O'Connor, 2011). Only a few studies have previously found evidence to suggest that this is possible. For example, Huisman, Jonker, Zonneveld, and Weissing (1999) showed that the ability of isolated algae species to survive on the lowest light level determined their competitive success in mixed communities. Bestion, García‐Carreras, Schaum, Pawar, and Yvon‐Durocher (2018) showed that phosphorus uptake rates of monocultures at different temperatures correctly predicted the competitive outcome between pairs of 6 phytoplankton species in the majority (71%) of the cases.

Simultaneous changes in multiple abiotic factors, such as those predicted to occur under climate change (e.g., rising sea surface temperatures in conjunction with changes in nutrient inputs), may also complicate predictions, as abiotic factors may interact (e.g., antagonistic or multiplicative), and organisms may respond in a way that is not accounted for from the responses to gradients of individual factors operating in isolation (Harley et al., 2017; Thomas et al., 2017). For instance, nutrient concentration was shown to interact with temperature to influence phytoplankton growth rates (Rhee & Gotham, 1981) and the cardinal temperatures (e.g., thermal optimum) of thermal performance curves (TPCs) (Bestion, Schaum, & Yvon‐Durocher, 2018; van Donk & Kilham, 1990; Thomas et al., 2017). In particular, N limitation can significantly lower the thermal optimum of the diatom *Thalassiosira pseudonana* toward colder temperatures (Thomas et al., 2017). Given this shift in thermal performance due to nutrient limitation, *T. pseudonana* may become less competitive in warm N‐limited waters. In contrast, *Phaeodactylum tricornutum* is known to be a good competitor for inorganic nitrogen when this nutrient is scarce because it can take up nitrate when abundant and store it for times of depletion (Cresswell & Syrett, 1982). Thus, the outcome of interspecific competition for inorganic nutrients might be expected to depend on temperature if the interaction of nutrient limitation with temperature is species‐specific. For example, *P. tricornutum* has been found to outcompete *T. pseudonana* and other species in maricultural ponds (Nelson, D’Elia, & Guillard, 1979), despite not being a dominating species in marine phytoplankton communities (Guillard & Kilham, 1977), which may be a reflection of nutrient availability or temperature characteristics in different systems. An early study on the competition between *P. tricornutum* and *T. pseudonana* showed that competition is indeed temperature‐dependent and that the thermal environment influences the competitive outcome in stationary phase cultures (Goldman & Ryther, 1976). Specifically, *P. tricornutum* was found to be a good invader and dominant species under cold to intermediate temperatures (<20°C), whereas it could not establish itself as an invasive species in warmer temperatures.

Here, the interaction of temperature and nitrate availability on direct competition between *P. tricornutum* and *T. pseudonana* was investigated along a temperature gradient. To test whether temperature and nutrient status have an interactive effect on the outcome of competition, experiments were conducted in both nutrient‐replete high N conditions and low N conditions (whereby nitrate limited yield). Specifically, it was hypothesized that (a) the species with the higher growth rate or carrying capacity at a given temperature and nutrient level as a monoculture would have the competitive advantage in mixed cultures and that (b) greater absolute differences in growth rate between species at a specific assay temperature would determine how quickly the poorer competitor would be displaced.

## 2 MATERIAL AND METHODS

Stock cultures of *P. tricornutum* (CCMP 2561) and *T. pseudonana* (CCMP 1335) were maintained in the University of Essex algal culture collection at 15.5 ± 1.0°C, in F/2 medium (Guillard & Ryther, 1962), prepared in artificial sea water (Berges, Franklin, & Harrison, 2001; Harrison, Waters, & Taylor, 1980), and grown on a 12:12‐hr light and dark cycle at a photosynthetic photon flux density (PPFD) of approximately 60 µmol photons m^−2^ s^−1^, which is close to optimum light levels for *P. tricornutum* (Geider, Osbonie, & Raven, 1986), and approximately 30% of the optimum light level for *T. pseudonana* (Geider, MacIntyre, & Kana, 1997; Geider, Maclntyre, & Kana, 1998). Stock cultures were transferred monthly as 1:36 dilutions.

### 2.1 Competition experiments

#### 2.1.1 High N competition experiment

Prior to experimentation, *P. tricornutum* and *T. pseudonana* were maintained in exponential growth for 2 weeks (approximately 10–14 generations) under stock culture conditions at 15.5 ± 1.0°C by transferring cultures into fresh F/2 medium once cell densities reached 500,000 cells/ml for *P. tricornutum* and 250,000 cells/ml for *T. pseudonana* (equivalent to about 5% of each species' carrying capacity).

Experiments using an aluminum temperature‐gradient block were conducted to assess (a) thermal performance curves (TPCs) for growth rate of monocultures of the two diatoms and (b) temperature dependence of competition between these species. The aluminum block was heated at one end and cooled at the other to generate a temperature gradient from 8.8°C at the cold end to 35°C at the hot end. The block provided 4 rows with 17 columns (assay temperatures) in each row. Temperature within each column was controlled to within 0.2°C, providing approximately 1.5°C increments along the temperature gradient. Illumination was provided from below by light emitting diodes (LEDs). PPFD was set to 150 ± 15 µmol photons m^−2^ s^−1^, and illumination was provided on a 12:12‐hr light and dark cycle.

**FIGURE 1:**
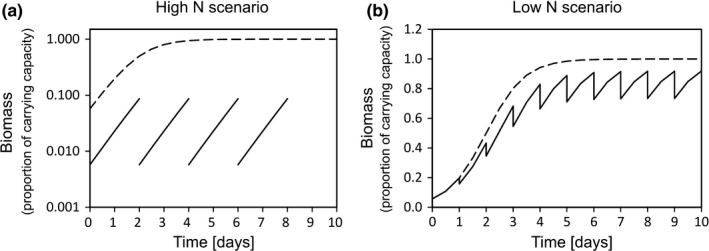
Simulation of semicontinuous cultures for obtaining and maintaining (a) nutrient‐replete (high N scenario) and (b) nutrient‐limited (low N scenario) cultures. Simulation assumes cultures are already acclimated to the nutrient‐replete growth under defined temperature, irradiance, etc. Assumes logistic growth with the nutrient‐replete growth rate = 1.4 d^−1^, and carrying capacity = 1. Dilution is approximately 10‐fold in the high N scenario and 1.25‐fold in the low N scenario. Solid lines are the culture density. Dashed line is the culture density that would be obtained in a batch culture undergoing logistic growth. Note that (a) is on a log scale to represent that high N exponential cultures were kept at low densities far from reaching stationary phase, whereas (b) is on a linear scale

Aliquots from stock cultures of each of the diatoms were used to inoculate 17 autoclaved borosilicate test tubes (5 ml assay volumes), which were distributed along the same 17‐assay‐temperature gradient described above. To assess the growth rate TPCs, in vivo chlorophyll *a* minimum fluorescence yield (*F*_0_) was used as a proxy for biomass (see section 2.2 below). Initial cell densities were approximately 160,000 cells/ml for *P. tricornutum* and 80,000 for *T. pseudonana*, corresponding to similar *F*_0_ values for both species. The monocultures were kept in exponential phase by diluting whenever an assay culture reached *F*_0_ values equivalent to cell densities corresponding to about 5% of each species' carrying capacity, approximately 500,000 cells/ml for *P. tricornutum* and 250,000 cells/ml for *T. pseudonana* (Figure 1a and Figure S1).

At the same time, to assess competition between these species, aliquots from the stock monocultures were combined to create a mixed culture with equal cell densities of 100,000 cells/ml in a 1:1 species ratio (50,000 cells of each species). The starting ratio could only be approximated, as cell densities were estimated from the relationship between cell density and *F*_0_ determined at 15.5°C, but fluorescence per cell is not constant as it varies between species and with assay temperature. This mixed culture was then used to inoculate 34 autoclaved borosilicate test tubes (5 ml assay volumes), which were distributed in duplicates along the same 17‐assay‐temperature gradient to assess the acute effect of temperature on competition. After mixed cultures had been established and distributed across the temperature gradient, 200 µl was removed daily from each mixed culture tube and preserved with Lugol's solution for cell frequency counts, that is, relative species abundances of the mixed cultures over time. 200 µl of fresh F/2 medium was added to keep the culture volume constant. As with the monocultures, *F*_0_ measured daily was used as an index of community biomass. The mixed cultures were kept in exponential growth by diluting when *F*_0_ reached values corresponding to about 5% of the carrying capacity.

After a further 2 weeks, the monocultures from each assay temperature that had been used to assess the growth rate TPCs of the two species were mixed together in an equal cell density ratio to investigate the effect of thermal acclimation on competition. Here, 2 mixed cultures to monitor competition were created at each assay temperature and aliquots were sampled daily to monitor changes in relative species frequencies.

Following completion of the competition experiment, triplicate monocultures of each species were grown across the gradient to increase the total replication for determining the TPC for growth rate of each monoculture to *n* = 4. However, in the final analysis, one of the *T. pseudonana* growth replicates was omitted as an outlier because the TPC was significantly different to the remaining three replicates (Figure S2a).

The above experimental design enabled the examination of (a) the TPCs for growth of *P. tricornutum* (*n* = 4) and *T. pseudonana* (*n* = 3) and (b) the acute interspecific competition along the temperature gradient in the 2 weeks following the transfer from their 15.5 ± 1.0°C source environment (*n* = 2) and of cultures acclimated for 2 weeks to temperatures of 8.8–35°C (*n* = 2).

#### 2.1.2 Low N competition experiment

Cultures were acclimated as a batch culture for 2 weeks in low N F/2 medium (starting concentration 55 µM in comparison to 882 µM in full F/2). As shown in Figure S3, this NO_3_ concentration reduced carrying capacity and ensured that cultures would reach the stationary growth phase at cell densities that did not saturate the fast repetition rate fluorometry (FRRf) signal. After acclimation, two monoculture replicates of each species were inoculated from early stationary phase stock cultures and grown in 5‐ml volumes across the 17 assay temperatures in the temperature‐gradient thermoblock (9.1–33.8°C) in the low N (55 µM) F/2 medium. The light regime was kept consistent with that used for the high N competition experiment and once again set to a PPFD in the range of 150 ± 15 µmol photons m^−2^ s^−1^ on a 12:12‐hr light and dark cycle.

The cultures were maintained in semicontinuous growth by removing 20% of the culture volume (1 ml) daily (used for determining cell abundance and nitrate concentration) and replacing this volume with fresh medium to maintain a total volume of 5 ml. This way cultures could be grown until cell abundance (yield) was limited by the concentration of nitrate provided in the growth medium and daily population growth rate was set by the dilution rate (Figure 1b). Although the daily sampling regime was different from the high N scenario to account for the necessities of culturing microorganisms in nutrient‐limited conditions, the results of the two nutrient scenarios can be compared as the same species were used and cultures were treated otherwise equally during the competition experiment.

The removed 1 ml samples were centrifuged at 5,000 *g* for 7 min at 4°C. A subsample of the supernatant (300 µl) was transferred to a flat‐bottom 96‐well plate (Thermo Scientific Nunclon, USA) and stored at −20°C to measure the sum of nitrate plus nitrite (NO_X_) at a later time (see section 2.4 below). The cell pellet was resuspended in the remaining volume (700 µl), fixed with formaldehyde (final concentration 1%), and stored at −20°C until cell abundances were estimated with flow cytometry (see section 2.5 below).

After growing in monocultures for 2 weeks, inorganic nitrogen concentrations in all treatments were depleted below the detection limit of the NO_x_ assay (i.e., <0.5 µM; see Figure S4). At this point, the fluorescence signals had stabilized or were declining, indicating that the yield was N‐limited (see Figure S5). Following this determination, one of the two monocultures of each species at each assay temperature was used to inoculate two mixed cultures for the competition phase of the experiment. Each mixed culture tube received 2 ml assay volumes from each monoculture, for a total volume of 4 ml, which was then topped up with 1 ml of low N F/2 medium to maintain a constant assay volume of 5 ml (competition replicates across assay temperatures at this point in time *n* = 2). The need to use 2 ml assay volume of each monoculture to not break with the 20% daily dilution had the consequence that the desired 1:1 species ratio at the start of mixed cultures could not always be reached. As in the high N experiments, 200 µl of the sampled volume was preserved daily with Lugol's solution for cell frequency counts. The remaining 800 µl volume was treated the same way as low N monocultures, with samples being centrifuged, and 300‐µl aliquots stored for NO_x_ analysis.

One week later, the second N‐limited semibatch monoculture replicate of each species was used to inoculate another set of two mixed cultures tubes at each assay temperature for a second duplicate competition experiment (increasing the amount of competition replicates to *n* = 4). Mixed cultures were kept in the same semicontinuous regime as monocultures with 20% of sample volume being harvested and replaced with fresh medium on a daily basis.

The above experimental design enabled the examination of (a) the thermal niche of *P. tricornutum* (*n* = 2) and *T. pseudonana* (*n* = 2) under low N conditions and (b) the acute interspecific competition along the temperature gradient at the onset of N limitation (*n* = 2) and of cultures that were cultured in isolation for an additional 7 days at 9.1–33.8°C (*n* = 2).

### 2.2 Chlorophyll fluorescence

In vivo chlorophyll *a* minimum fluorescence *F*_0_ (minimum fluorescence yield), used as a proxy for biomass, and photosynthetic efficiency *F_v_/F_m_* were measured daily via FRRf with a FastTracka II fluorometer and Fast Act laboratory system (both CTG Ltd). Photosynthetic efficiency was calculated via the formula (*F_m_* − *F*_0_)/*F_m_* (*F_m_* = maximum fluorescence yield) by the FastPro software (version 1.0.55) used to attain and record FRRf measurements. Peak excitation was at 435 nm and fluorescence emission measured at 680 nm (with a 25 nm bandwidth). The measuring protocol was set to 24 sequences per acquisition with a 100 ms sequence interval and a 20 s acquisition pitch.

Measurements were taken on cells that were dark acclimated for 30 min at their assay temperature, and profiles of fluorescence emission were fitted within the Fast Pro 8 software (version 1.0.55) (Chelsea Technologies).

### 2.3 Mixed population species frequency counts

Frequency counts for the high N and low N experiment were carried out to monitor the relative abundance of each species in the mixed populations over time. Flow cytometry could not be used to discriminate between the two species as their forward scatter (FSC) and chlorophyll *a* fluorescence overlapped. As such, Lugol's preserved cells were allowed to settle to the bottom of the well‐plate wells (approximately 3 hr) before frequency counts were performed using an Olympus inverted microscope at 200× magnification. In each fixed sample, a minimum of 400 cells was counted in order to calculate a *P. tricornutum‐*to‐*T. pseudonana* cell abundance ratio.

### 2.4 NO_x_ assay (total nitrite/nitrate analysis)

NO_x_, the sum of nitrite and nitrate, concentration was measured using the method of Schnetger and Lehners (2014) to track the monocultures to nitrogen depletion before mixing them together for the low N competition experiment. The 300 µl frozen aliquots were defrosted on ice, and a 150 µl volume was transferred to a fresh 96 well‐plate, and 75 µl of the NO_x_ reagent was added. The reagent was mixed with the assay volume by pipetting up and down several times and incubated at 45 ± 5°C for 60 min before measuring absorbance at 540 nm in a FLUOstar Omega plate reader (BMG Labtech).

### 2.5 Flow cytometry

For the low N experiment only, flow cytometry data were used to confirm that cell densities had stabilized, despite *F*_0_ fluorescence declining, as cell physiology continued to adjust to N limitation. Cellular chlorophyll content is known to decline under N limitation (Parkhill, Maillet, & Cullen, 2001), and as a consequence, the ratio of *F*_0_ to cell density will also decline (Figure S6). Because cellular physiology was stable in exponentially growing cultures of the high N experiment, flow cytometry was not required.

Previously collected samples (as described above in section 2.1.2) were defrosted on ice and quantified with an Accuri C6 flow cytometer (BD Biosciences) equipped with a blue laser (488 nm). Diatom populations were discriminated based on chlorophyll *a* fluorescence (>670 nm) and forward scatter (FSC). FSC was used as a proxy for cell size to observe potential changes across assay temperatures after 15 days of monoculture growth in the low N medium. After counting cells within 45 µl of sample volume per sample, population statistics were calculated using particle counts within gates with the supplied BD Accuri C6 Analysis Software (Version 1.0.264.21).

### 2.6 Data analysis

#### 2.6.1 Growth rates and carrying capacities

Growth rates (*µ*) in high N medium (starting nitrate concentration of 882 µM) were calculated for each monoculture replicate and dilution phase as the slope of the linear regression of the natural log of *F*_0_ over time (Figure S1). The growth rate calculated for the 1st dilution phase was not used for further analysis as the cultures had not yet acclimated to assay conditions. Growth rates from all subsequent dilution phases were used to fit the TPC models (see section 2.6.2 below).

The initial exponential growth rates during the nutrient‐replete phase in low N semicontinuous cultures (with a starting nitrate concentration of 55 µM) were calculated using FRRf data from the first 4 days of culturing before the exponential increase in *F*_0_ slowed (see Figures S5 and S7). Growth rates were calculated with Equation (1) as.

(1) $$\mu =k+\ln\left(\frac{1}{0.8}\right)$$

where *k* is the slope of ln(*F*_0_) over time, and $\ln\left(\frac{1}{0.8}\right)$ (natural logarithm of 100% medium (i.e., 1.0) over 80% medium (i.e., 0.8)) was added to *k* to account for the dilution by the daily volume removed (1 ml) from the 5 ml culture.

Cell densities increased to a maximum by day 5 that was sustained thereafter (see Figure S8). The carrying capacity, K, under the imposed dilution rate of 0.22 d^−1^, was calculated for each replicate and at each assay temperature from the average cell densities measured after experimental day 4.

#### 2.6.2 Thermal performance curves

All twelve equations available in the R package “temperatureresponse” (Low‐Décarie et al., 2017) were fitted to the temperature‐dependent data of growth and carrying capacities obtained from monocultures of *P. tricornutum* and *T. pseudonana* (Table S1). For the growth model fitting in high N cultures, data from all dilution phases, except dilution phase 1, which was regarded as the growth phase in which the cultures acclimated to assay conditions, were used. The average growth rate across dilution phases was calculated for each species and replicate at each assay temperature, and the temperature dependence was modeled based on these average growth rates. For the low N scenario, thermal performances were modeled on the acute growth rates obtained from the first 4 days of low N growth (Figure S7) and on the thermal response of carrying capacities across assay temperatures (Figure S8).

The most appropriate equation for each species was selected based on a visual inspection of the data in combination with Akaike's information criterion (AIC) values (see Figure S9). Visual inspection was necessary as AIC values alone did not always predict the equation that best described the temperature‐dependent data, especially at suboptimal temperatures. For *P. tricornutum*, Equation (2) from Ratkowsky, Lowry, McMeekin, Stokes, and Chandler (1983) was found to be the best model across all replicates.

(2) $$\text{Rate}={\left[a\cdot \left(T-T_{\min}\right)\right]}^{2}\cdot {\left[1-\exp\left(b\cdot \left(T-T_{\max}\right)\right)\right]}^{2}$$

For *T. pseudonana,* Equation (3) from Montagnes, Morgan, Bissinger, Atkinson, Weisse (2008) was found to best describe the thermal performance.

(3) $$\text{Rate}=a+b\cdot T+c\cdot T^{2}$$

where *T* in both equations is the assay temperature at which growth rate and carrying capacity were measured.

The fitted TPCs were used to obtain the optimum temperature for growth (*T_opt_*), the maximum growth rate (*µ*_max_) at this temperature, and the low and high temperatures at which *μ* = 0.5*μ*_max_ (CT_50min_ and CT_50max_). Single parameters of nonlinear model fits can be found in Tables S2‐S4.

To investigate whether the thermal niche differed significantly between (a) the two species, and (b) two nutrient scenarios, a two‐way MANOVA was used to examine the 4 dependent variables (*T_opt_*, CT_50min_, CT_50max_, and *µ*_max_). The two independent variables were “species” (levels: *P. tricornutum* and *T. pseudonana*) and “nutrient scenario” (levels: high N and low N). Sample size was *n* = 11 (4 *P. tricornutum* replicates in high N, and 2 in low N, as well as 3 *T. pseudonana* replicates in high N and 2 in low N), and significance levels were set to a p‐value of 0.05. By conducting a MANOVA, we could access all pairwise comparisons to determine which cardinal temperatures (a) changed significantly in the overall thermal niche and (b) were affected by a “species” and “nutrient scenario” interaction.

To test whether the thermal dependency of carrying capacities under low N conditions differed between the two species, individual one‐way ANOVAs were conducted with “species” as the independent variable on the four dependent parameters *T*_opt_, CT_50min_, CT_50max_, and maximum carrying capacity (*K*_max_). MANOVA testing was not possible due to the replication number of *n* = 2 for the monocultures grown in low N conditions. *p*‐values of the single ANOVAs were adjusted for multiple testing with a Bonferroni correction and reported as *q*‐values.

#### 2.6.3 Predicted competition coefficients

Competition coefficients across assay temperatures in high N and low N conditions were predicted from the mean growth rates (Equation 4a) or mean carrying capacities (Equation 4b) of each species calculated from the fitted TPCs.

(4a) $$\text{Predicted competition coefficient}\;\left(T\right)={\left(\mu_{1}\right)}_{T}-{\left(\mu_{2}\right)}_{T}$$

where (*µ*_1_)*_T_* and (*µ*_2_)*_T_* are the growth rates of species 1 and species 2 at temperature *T*.

(4b) $$\text{Predicted competition coefficient}\;\left(T\right)={\left(K_{1}\right)}_{T}-{\left(K_{2}\right)}_{T}$$

where (*K*_1_)*_T_* and (*K*_2_)*_T_* are the carrying capacities of species 1 and 2 at temperature *T*.

Since competition coefficients are traditionally calculated from growth rates (e.g., Low‐Décarie, Fussmann, & Bell, 2011; Segura et al., 2011), coefficients calculated from carrying capacities must be treated with caution, as the relationship between K and competitive outcome is not established for steady‐state nutrient‐limited conditions. Higher K does not necessarily signify better competition ability. Under steady‐state nutrient limitation, it is R* that determines competitive outcome (Tilman, 1981), but because the NO_X_ concentrations were below the detection limit of our assay, we could not measure R* in our experiment.

The predicted temperature dependence of competition coefficients was modeled with a local estimated scatter plot smoothing (LOESS) from the R core package “stats” (R Core Team, 2016). LOESS fits a smoothing curve into data that is distributed on a scatter plot to graphically represent the relationship between an independent and a dependent variable. It is a suitable method for visualizing complex nonlinear relationships. The final smoother curve is the result of many local regression curves fit together (Isnanto, 2011; Jacoby, 2000). As such, it is a tool for predicting specific points on a regression, such as the inflexion point of competition, and to explore data. To display the strength of how closely our LOESS smoother followed the calculated competition coefficients, we reported *R* ^2^‐values and residual standard errors (RSE) in the results.

#### 2.6.4 Observed competition coefficients

Changes in the ratio of the abundances of the two species through time (Figures S10 and S11) were used to calculate observed competition coefficients. The competition coefficient was calculated as the slope of the change in the natural logarithm of species frequency over time. Competition coefficients were calculated with Equation (5) for mixed high N cultures and mixed low N cultures.

(5) $$\text{Competition coefficient}=\frac{\ln\left(\frac{N_{P.tricornutum}:N_{T.pseudonana}\left(t\right)}{N_{P.tricornutum}:N_{T.pseudonana}\left(0\right)}\right)}{t}$$

where *N* = species abundance, and *t* = time. At the temperature at which competition coefficients are 0, stable coexistence of the two species is expected.

As described above (Section 2.1), each nutrient scenario had 4 competition replicates across the temperature gradient. In the final analysis of competition under high N conditions, the acute and acclimated competition replicates were pooled because we did not find an effect of temperature acclimation and could not observe a significant difference in the temperatures at which the competitive advantage switched from *P. tricornutum* to *T. pseudonana* (*F*_1,2_ = 4.34, *p* = .173) (Figure S12, and Table S5). Due to a lack of an effect of temperature acclimation on the progression of competition across temperatures, we did not structure the competition replicates into acclimated and nonacclimated in the low N scenario and focussed on culturing the monocultures to N limitation, pooling the four competition replicates as well.

In order to determine whether nutrient regime significantly changed the temperature at which *P. tricornutum* and *T. pseudonana* coexisted (i.e., the inflexion point temperature at which the competition coefficient equalled 0), we conducted a one‐way ANOVA with the temperature of competition inflexion as the response variable and “nutrient regime” as the independent variable. Significance levels were set to a *p*‐value of .05.

All data analysis and calculations mentioned above were carried out with the statistical software R (version 3.3.1) (R Core Team, 2016).

## 3 RESULTS

### 3.1 Differences in temperature and nutrient response between species in monoculture

**FIGURE 2:**
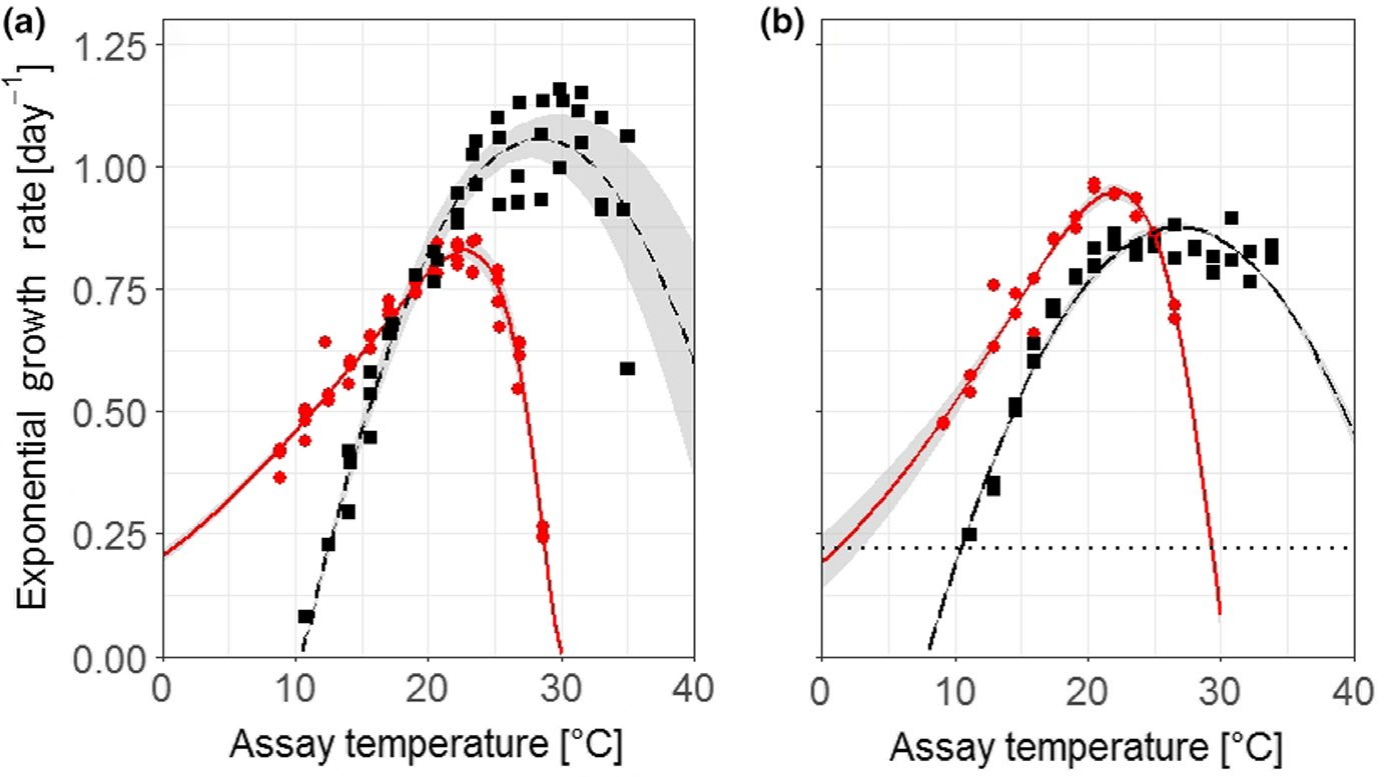
Average temperature response curves depicting (a) acclimated growth rates of *Phaeodactylum tricornutum* (red circles and solid line) and *Thalassiosira pseudonana* (black squares and dashed line) under high nitrate conditions (*n* = 4 and 3 respectively) and (b) the initial acute growth rate (days 0–4) under low N conditions (*n* = 2 for both species). Shaded area denotes standard deviation of the average model calculated from replicate model fits. Symbols in (a) represent the growth rates from distinct biological replicates and were calculated from the average growth rates across dilution steps after the diatoms had acclimated to growth under assay conditions. Dotted line in (b) indicates ln(1/0.8), the daily dilution rate that was imposed by the sampling regime in these semicontinuous cultures; cell abundance would have declined in cultures with growth rates below this value which equates to a growth rate of 0.22 d^−1^. Raw data used to calculate growth rates in the high nitrate (882 μM ${\text{NO}}_{3}^{-}$ ) medium (a) can be seen in Figure S1, and data used to calculate growth rates in the low N (55 μM ${\text{NO}}_{3}^{-}$ ) medium (b) can be seen in Figure S7. Nonlinear model outputs of the single replicate models used to calculate the average model can found in Tables S2 and S3, and single model fits on the individual growth replicates can be found in Figure S2a,b

**FIGURE 3:**
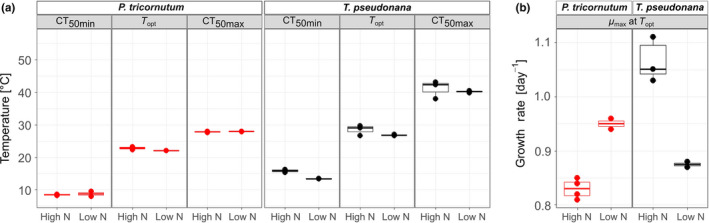
Comparison of (a) cardinal temperatures and (b) maximum growth rates from thermal performance curve model fits for growth rates between *Phaeodactylum tricornutum* and *Thalassiosira pseudonana* under high N and low N growth conditions. For *Phaeodactylum tricornutum,* *n* = 4 in high N and *n* = 2 in low N. For *Thalassiosira pseudonana,* *n* = 3 in high N and *n* = 2 in low N

When *P. tricornutum* and *T. pseudonana* were grown as monocultures, the thermal niches of the two species differed significantly from one another irrespective of nitrogen conditions (2‐way‐MANOVA, *F*_1,7_ = 103.49, *p* < .001) (Figure 2, Table S6). The contrasting thermal niches between the two species were evident in differing cardinal temperatures (CT_50min_, *T_opt_*, CT_50max_) (Figure 3a) and *µ*_max_ (Figure 3b). *Phaeodactylum tricornutum* occupied a cooler thermal niche than *T. pseudonana*, characterized by lower cardinal temperatures (Figure 3a), and could not sustain growth when temperature was in excess of 30°C (Figure 2). In contrast, *T. pseudonana* occupied a warmer thermal niche and was struggling to grow toward the coolest tested assay temperatures. In the range of 10°C and below, the growth rate of *T. pseudonana* approached 0 d^−1^, indicating that the thermal tolerance minimum of this *T. pseudonana* strain was being reached (Figure 2).

N limitation altered the thermal niche of both species (Figures 2 and 3). The 2‐way MANOVA confirmed that nitrate levels had a significant effect on the cardinal temperatures of the TPCs of the two monocultures (*F*_1,7_ = 9.59, *p* < .05). However, the two diatoms responded differently to a reduction in N and consequently there was a significant interaction between “N regime” and “species” (*F*_1,7_ = 17.84, *p* < .01). Specifically, the thermal niche for *P. tricornutum* became slightly more narrow (increase in CT_50min_ by 0.31°C), and *µ*_max_ at *T_opt_* increased by 14%. In contrast, the thermal niche of *T. pseudonana* widened (CT_50min_ dropping by 2.38°C), and the *µ*_max_ of *T. pseudonana* getting reduced by 17% (isolated ANOVA results within the MANOVA test for each cardinal temperature can be found in Tables S7‐S10).

**FIGURE 4:**
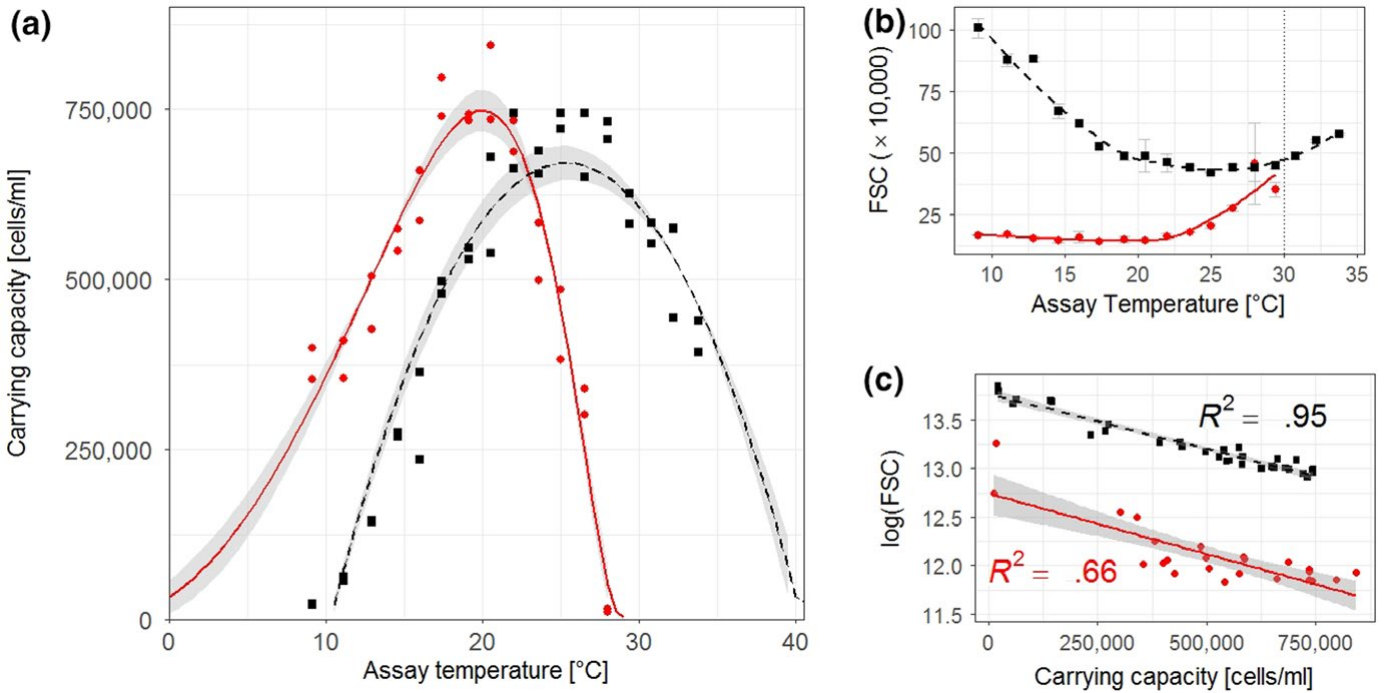
(a) Temperature response curves of carrying capacity of *Phaeodactylum tricornutum* (red circles and solid line) and *Thalassiosira pseudonana* (black squares and dashed line) under nitrogen depleted conditions in the N‐limited phase of semicontinuous culturing in the low N medium (*n* = 2 for both species). Shaded area denotes standard deviation of the average model calculated from replicate model fits. Symbols show the carrying capacities from distinct biological replicates across the assay‐temperature range. Raw data used to calculate carrying capacities can be seen in Figure S8. Nonlinear model outputs of the single replicate models used to calculate the average model can be found in Table S4, and single model fits on the individual growth replicates can be found in Figure S2c. (b) Forward scatter (FSC) from flow cytometry data of the low N experiment across assay temperatures. FSC measurements depicted here were taken on the last day of replicate monocultures before they were mixed together for the competition experiment. The purpose of the solid and dashed LOESS lines is to visualize the U‐shaped trend of the data across assay temperatures (*R* ^2^ = 0.85, and RSE = 4.503 for *Phaeodactylum tricornutum*; *R* ^2^ = 0.97, and RSE = 3.378 for *Thalassiosira pseudonana*). LOESS was not used as a model to predict the relationship between temperature and cell size. Dotted line at 30°C indicates the critical maximum temperature for *Phaeodactylum tricornutum* beyond which it could not sustain growth. (c) Correlation between carrying capacity K and log(FSC) for both species. Solid and dashed lines are linear models of the regression

**FIGURE 5:**
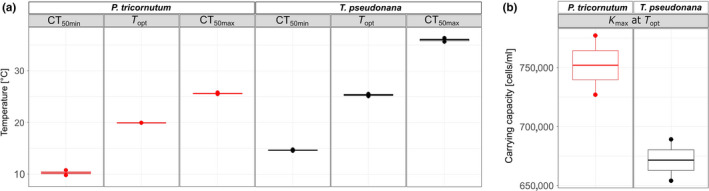
Comparison of (a) cardinal temperatures and (b) maximum carrying capacities from thermal performance curve model fits for carrying capacities between *Phaeodactylum tricornutum* and *Thalassiosira pseudonana* in low N conditions (*n* = 2 for both species)

Similar to the growth rate results, carrying capacities also displayed a unimodal nonlinear response along the assay‐temperature gradient and monocultures reached higher carrying capacities the closer the assay temperatures were to the species' *T_opt_* (Figure 4a). All of the cardinal temperatures for carrying capacity TPCs differed significantly from one another between species (*T_opt_*: *F*_1,2_ = 545.9, *q* < 0.01; CT_50min_: *F*_1,2_ = 78.28, *q* = 0.05; CT_50max_: *F*_1,2_ = 743.1, *q* < 0.01) (Figure 5a). Similar to the growth trends in the high N scenario, these significant differences arose because the thermal niche of *T. pseudonana* was shifted more toward warm temperatures than *P. tricornutum*. Despite differences in the thermal niches, the maximum carrying capacity at *T_opt_* did not differ significantly between species (*F*_1,2_ = 6.96, *q* = 0.48) (Figure 4b). Individual test statistics for the single one‐way ANOVAs can be found in the Supplementary Material (Tables S11‐S14).

After 15 days of growing the monocultures in the low N medium, cell size showed a U‐shaped trend across assay temperatures with cell size increasing toward the coldest and warmest tested assay temperatures (Figure 4b). There was a strong negative linear correlation between the logarithm of cell size and K (Figure 4c), with the smallest cell sizes being observed at assay temperatures closest to each diatom's *T_opt_*, meaning that cell size increased toward the diatoms' physiological limits.

### 3.2 Competition across assay temperatures

**FIGURE 6:**
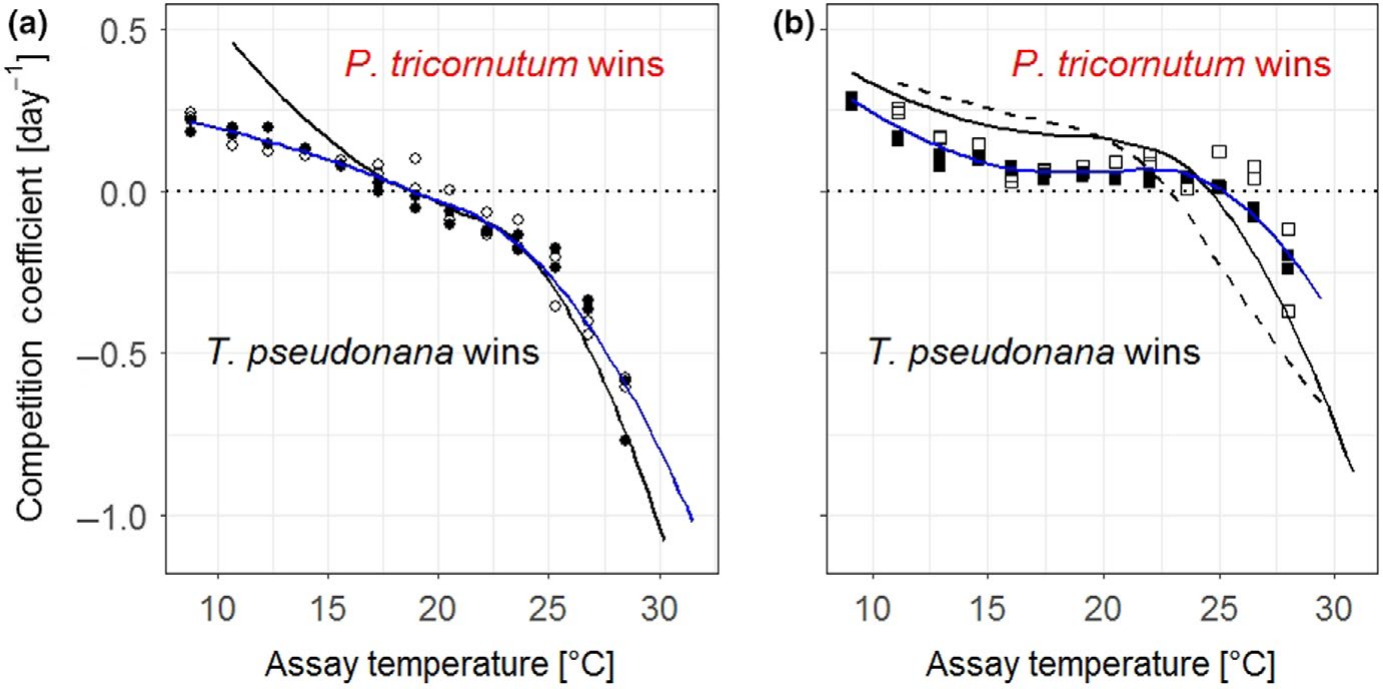
*Predicted (black lines) and observed (symbols) competition coefficients between Phaeodactylum tricornutum and Thalassiosira pseudonana across assay temperatures in (a) high N conditions and (b) low N conditions. A coefficient of 0 indicates no competitive advantage for either species and predicts stable coexistence under the assay conditions. At coefficients greater than 0, Phaeodactylum tricornutum has the competitive advantage, and at coefficients below 0, Thalassiosira pseudonana has the advantage. The further the competition coefficient deviates from 0, the stronger is the competition between the two species. In (a), the solid black line indicates predictions for competition coefficients made from high N monoculture growth rates. The solid blue line is the LOESS smooth to visualize the progression of all observed high N competition coefficients across temperatures (R^2^ = 0.97, RSE = 0.047). Observed coefficients were calculated from changes in species frequencies over time in mixed cultures (data from Figure* S10 *), calculated with Equation (* 5 *). Closed symbols represent the competition coefficients that were started when stock cultures were transferred to the temperature‐gradient block, open symbols those that were started 2 weeks later to investigate the potential effects of temperature acclimation. In (b), solid black line indicates predictions made from the initial exponential growth rate in low N medium for monocultures, whereas the dashed line indicates predictions made from monoculture carrying capacities across the temperature gradient. The solid blue line is the LOESS smooth to visualize the progression of all observed low N competition coefficients across temperatures (R^2^ = 0.86, RSE = 0.051). Observed coefficients were calculated from changes in species frequencies over time in mixed cultures (data from Figure* S11 *), calculated with Equation (* 5 *). Closed symbols represent competition replicates that were started at the onset of N limitation, and open symbols those that were started from monocultures that were cultured under N limitation for an additional 7 days*

Monoculture TPCs were used to predict competition across assay temperatures. Under high N conditions, LOESS (represented by the solid black line in Figure 6a, *R* ^2^ = 0.998, and RSE = 0.021) predicted an inflexion point at 18.8°C (i.e., a competition coefficient equal to 0), indicating that *P. tricornutum* was predicted to outcompete *T. pseudonana* from the coldest assay temperature up to 18.8°C. N limitation was predicted to alter the competition across assay temperatures whereby the inflexion point would shift horizontally across the temperature axis to 24.6°C (5.9°C warmer than under high N conditions; solid black line in Figure 6b, *R* ^2^ = 0.996, and RSE = 0.026). Because K also showed a response across the assay‐temperature gradient, it was possible to make a second prediction of competition in the low N scenario based on K. Competition coefficients calculated from K predicted the switch at 22.9°C (4.2°C warmer than under high N conditions; dotted black line in Figure 6b, *R* ^2^ = 0.992, and RSE = 0.039). Competition was predicted to be strongest at assay temperatures where the growth rates or carrying capacities in monocultures exhibited greatest divergence between species. Naturally, a species would lose the competition at an assay temperature where it could not sustain growth as an isolated species, having reached its physiological limits. Between the upper and lower temperature thresholds where both species could grow, the competitive outcomes were determined by the thermal growth performances of the species in isolation.

When the two diatoms were mixed together and competed against one another, the observed competition coefficients displayed a similar progression across assay temperatures as the predictions. In high N conditions, inflexion points occurred at 18.8 ± 1.2°C (predicted at 18.8°C) (Figure 6a). As predicted, *P. tricornutum* won competitions across temperatures colder than the inflexion point, whereas *T. pseudonana* won competitions at temperatures warmer than the inflexion point. When the nitrate concentration was reduced, the competitive switch occurred at a warmer temperature, as predicted from the temperature dependencies of low N growth rates and carrying capacities (Figure 6b). The average temperature for the inflexion point in low N medium was 6.5°C greater than in high N medium and differed significantly (*F*_1,6_ = 83.11, *p* < .001) (Table S15). The inflexion point was located at 25.3 ± 0.6°C (predicted at 24.6°C when growth rate was used to calculate the competition coefficients or 22.9°C when carrying capacity was used for the prediction). Pooling of mixed culture replicates for the final analysis due to a lack of a temperature acclimation signal in the high N scenario did not affect our interpretation of the conclusion that the competitive shift occurred at higher temperatures in the low N scenario.

### 3.3 Predictability of change in competitive ability

In both nutrient scenarios, the predicted and observed competition coefficients were strongly correlated (*R* ^2^‐values of 0.76–0.98), indicating that competition coefficients were very predictable from growth in monocultures (Table 1).

**TABLE 1:** Parameters for linear models (*y* = *ax *+* b*) correlating predicted (*x*) and observed competition coefficients (*y*) across assay temperatures

Correlation	Linear model parameters					
	*R* ^2^ of correlation	Slope ± 95%‐confidence interval	*y*‐Intercept ± 95%‐confidence interval	*df*	*t*‐value	*p*‐value of correlation
High N growth rates with competition coefficients (*n* = 13)	0.98	1.37 ± 0.13	0.06 ± 0.04	11	22.60	<.001*
Low N growth rates with competition coefficients (*n* = 14)	0.93	1.73 ± 0.29	−0.01 ± 0.05	12	13.09	<.001*
Low N carrying capacity with competition coefficients (*n* = 13)	0.76	1.96 ± 0.72	−0.05 ± 0.11	11	5.98	<.001*

Note

Significant correlations (alpha level of 0.05) that conclude a relationship between predictions and observations are marked in bold and with an asterisk.

Progression of predicted and observed competition coefficients across temperatures was very similar, yet deviations existed between predictions and observations. For example in high N conditions, observed coefficients tended to be lower than predictions across the five lowest assay temperatures, and therefore, observed competition was not as strong as predicted across the assay temperatures where *P. tricornutum* outcompeted *T. pseudonana* (competition coefficient > 0). Likewise, across the temperatures at which *T. pseudonana* was predicted to win (competition coefficient < 0), the strength of the competition coefficients was slightly overestimated and predicted to be more negative than observed values (Figure 6a).

In low N conditions, the predicted and observed coefficients aligned less strongly than in high N conditions, but correlation was still high (see *R* ^2^ in Table 1). Predictions were more accurate when they were made from low N growth rates than from carrying capacities (Figure 6b). As with the high N experiment, the predicted coefficients were typically stronger than the observed. In addition, both low N predictions underestimated the temperature of the inflexion point and predicted the competitive advantage to switch at a lower assay temperature than it did in the experiment, therefore, underestimating the temperature range at which *P. tricornutum* was the better competitor.

## 4 DISCUSSION

In this study, competition between *P. tricornutum* and *T. pseudonana* was determined primarily by the growth performance of these species under specified thermal and nutrient regimes in isolation and indicated that they did not interact strongly in mixed populations. The results provide support for our hypotheses as we show that in relatively simple systems, prediction of competitive outcomes from isolated species performance across a thermal gradient is possible. The amount of N present in the mixed cultures influenced the competition across the temperature gradient and showed that alterations in nutrient concentrations have the potential to change the outcomes of competition with all else being constant. Further aligning with our hypothesis, the better growing species in isolation had the competitive advantage in mixed cultures, with the switch in competitive advantage occurring at or close to the temperature where the TPCs of the two species intercepted. The poorer competitor also lost the competition quicker when the absolute differences between monoculture thermal performances were the largest.

### 4.1 Reduced nitrogen impacts competition across temperatures

Under low N conditions, we observed *P. tricornutum* to be the better competitor across most of the tested assay temperatures. Although N uptake rates were not measured in this experiment, the increased competitive success of *P. tricornutum* in low N conditions, and a warmer inflexion temperature of competitive advantage as a result thereof, could be attributed to its previously reported high affinity and high uptake rates for nitrogen (Goldman & Ryther, 1976; Grover, 1991; Sharp, Underhill, & Hughes, 1979). This may have facilitated *P. tricornutum* to win competitions across most of the N‐limited temperature gradient, including at assay temperatures up to 3.2°C warmer than predicted (22.9°C prediction from K across temperatures compared to warmest observed inflexion point in low N conditions at 26.1°C). To confirm that the increased competitive success can be explained through better nitrogen uptake rates and storage abilities, future experiments should quantify N uptake rates of the two species under the assay conditions described in this study. Its good nitrogen uptake ability could help *P. tricornutum* to win competitions under N‐limiting conditions at temperatures where other species can normally reach higher carrying capacities or grow faster when N is not limiting. However, the high N affinity of *P. tricornutum* may be nullified in conditions where N content of surface waters is high. For *T. pseudonana*, the reduction in *µ*_max_ and shift of *T_opt_* toward colder temperatures in low N conditions was in accordance with Grimaud, Mairet, Sciandra, and Bernard (2017) and confirmed the effect of nitrogen concentration on *T. pseudonana* growth rates. This change in thermal performance indicates that *T. pseudonana* may become less competitive in warm N‐limited waters, but its competitive ability should be reinstated following the introduction of N into a system, for example, in coastal up‐welling zones.

Despite presumed better nitrogen uptake abilities, *P. tricornutum* did not win all competitions across the whole assay‐temperature gradient in low N conditions. This may be due to the fact that good N uptake ability was offset toward the warm assay temperatures by physiological limits for growth of this diatom. Past research has identified R* across temperatures as a U‐shaped function (Lewington‐Pearce et al., 2019; Tilman, Mattson, & Langer, 1981), and warmer temperatures were found to increase nitrogen demand in phytoplankton (Toseland et al., 2013). Toward the growth limits, the minimum nutrient requirements for a species rise rapidly within a small temperature range as cells become stressed and need a larger internal nutrient content (cell quota) to survive while other physiological limits become more important (Rhee & Gotham, 1981; Thomas et al., 2017; Tilman, 1981). The circumstance that cell size was also found to display a U‐shaped trend with assay temperatures could have reinforced the observed patterns of competition across the assay‐temperature gradient. Cell size is known to play a role in competitive success and species dominance because larger, slower growing cells tend to have lower nutrient uptake rates relative to smaller cells with more beneficial surface‐area‐to‐volume ratios (Gallego, Venail, & Ibelings, 2019; Litchman & Klausmeier, 2008; Smith & Kalff, 1982). This study provides further support of this relationship, whereby cells growing toward their physiological limits (thermal extremes) were larger and grew slower, thus potentially amplifying the rate at which a species was losing competitions close to its physiological limits. As surface temperatures are expected to increase in aquatic systems worldwide, this temperature dependence of R* and cell size might play a crucial role for population dynamics under warming scenarios in future marine and freshwater ecosystems (Bernhardt, Sunday, & O'Connor, 2018; Lewington‐Pearce et al., 2019). Approaching physiological limits through warming might shift abiotic conditions in a direction in which a competitor might be at a loss although it is generally better at taking up nutrients in more moderate environments. Such an environmental change toward more critical conditions might offset the ability of a species to reduce nutrients to a concentration that is lower than the R* of a direct competitor (McPeek, 2019).

The observed changes in species frequencies over time in the low N scenario may have been due to interspecific differences in the capacity for surge uptake. Greater capacity for surge uptake is commonly defined as higher values of the maximum velocity of nutrient uptake (*V*_max_ in the Michaelis–Menten equation), induced in phytoplankton as a physiological adjustment to nutrient limitation (Morel, 1987). The species with the greater capacity to increase *V*_max_ will sequester nutrients quicker, effectively depriving them from the competitor with a lower capacity to increase *V*_max_. In our low N experiment, daily additions of fresh medium were followed by extensive phases of nutrient starvation when this added nitrate was fully taken up. Because we did not measure nitrate uptake kinetics, we cannot conclude from our experiments whether the outcome of competition depended on the ability to sequester nitrate quickly after N addition or the ability to live off the lowest nitrate concentrations (interspecific differences in the half saturation constant for uptake, *K_m_* of the Michaelis–Menten curve). The growth rates calculated from the increase in fluorescence during the first few days of the low N monocultures might be indicative of surge uptake playing a role in the competitive success under low N conditions. The predictions of competitive outcomes from the exponential growth phase in low N medium did align closer with the observed competitions than the predictions from carrying capacities (see Figure 6b). However, using fluorescence‐based growth rates or maximum K under low N conditions as predictors of competition can be regarded as proxies at best. Confirmation of a role for surge uptake in competition between these two species requires further experiments. The determination of N uptake rates and residual nitrate concentrations using more sensitive methods (e.g., stable isotopes) may help improve predictions of competitive success.

### 4.2 Predictability of competition under environmental change

In this study, monoculture responses of phytoplankton were found to be good predictors of competitive outcomes. Previously, they also have been identified as good predictors of phytoplankton biogeography (Barton, Irwin, Finkel, & Stock, 2016; Thomas, Kremer, Klausmeier, & Litchman, 2012). Whether such culture‐based measurements are ultimately suitable for forecasting shifts in phytoplankton communities in response to environmental change and whether the findings can be generalized for other species pairs or taxa remains to be tested. Predictability of competitive outcomes in complex communities still presents a major challenge (Pennekamp et al., 2019).

As the species in this study did not appear to influence one another in mixed cultures, it is likely that allelopathic interactions did not play a role in this competition scenario. Indeed, monoculture performance would cease to be a good predictor of competitive ability if one species had a toxic effect on the other in a two species competition. For example, dinoflagellates can inhibit growth of competitors by releasing toxins (e.g., Kubanek, Hicks, Naar, & Villareal, 2005), and some diatoms excrete chemicals that inhibit growth of other diatoms (Pichierri et al., 2017). A slower growing species might be able to gain a competitive advantage through the excretion of allelopathic substances upon sensing another species in its surrounding. Such species‐specific interactions may explain some of the uncertainty in modeling natural phytoplankton communities. Furthermore, TPCs for growth of the same species were found to vary slightly from laboratory to laboratory even when similar protocols were employed (Boyd et al., 2013). These differences could be driven in part by data quality and model fitting (Low‐Décarie et al., 2017), but they still raise concerns that predictability of competition response, which is dependent on thermal niche parameters and *µ*_max_, only remain valid within a specific setting. In addition, adaptive changes in nutrient requirements or physiological parameters could change competitive abilities and reduce predictability. Elucidating the role of evolutionary change and whether long‐term interactions are affected by genotypic variability will therefore play a crucial role in understanding future phytoplankton community structure (Bernhardt et al., 2020).

Our observations in monocultures were suitable for predicting the inflexion points of competition. However, the predictions across the whole temperature range commonly overestimated the magnitude of competition coefficients, and this increased the further the coefficients were from 0 (see Figure 6). The mismatch between predicted and observed coefficients at the extreme temperatures may be due to the fact that light microscopy counts could not distinguish between viable and nonviable cells. The outcome being that cell frequency counts overestimated the number of reproducing cells of the losing competitor and incorrectly tipping the competition coefficient in its favor. Future studies could employ the use of a cell viable stains to discriminate between live and dead cells (e.g., Baker et al., 2018) when conducting cell frequency counts.

### 4.3 Wider implications of the study

The nitrogen loads to many coastal waters are predicted to increase in the future due to agricultural runoff or other human activities (Beman, Arrigo, & Matson, 2005; Nixon, 1995), and surface waters are expected to become more stratified as warming increases (Bestion, Schaum, et al., 2018). The findings that competitive outcomes change across temperatures and are dependent on the amount of N present in the experimental system imply that environmental changes and alterations of marine environments will affect how phytoplankton communities will be structured in the future. Changes in species interactions could then have subsequent effects on ecosystem functioning since distinctively structured communities cycle nutrients and carbon differently or have varying nutritional value for higher trophic levels (Falkowski, Barber, & Smetacek, 1998; Litchman, Klausmeier, Miller, Schofield, & Falkowski, 2006; Schaum, Rost, Millar, & Collins, 2012).

Regardless of the importance for natural ecosystems, the finding that competitive outcomes could be well predicted in a simplified system could be of relevance for large scale pond maricultures or other algae biotechnological settings where simple model communities or monocultures are established for harvest or extraction of secondary metabolites. The ability to predict what the population composition would be under a defined set of abiotic parameters could help to control growth dynamics or purity of a culture (Regan & Ivancic, 1984). By being able to predict composition, parameters can be altered to potentially stabilize mixed cultures or purify them through changing temperature or nutrient loading.

## 5 CONCLUSION

The current study demonstrated that competition between two diatoms could be well predicted in a controlled laboratory system. The aquatic environments where these algae naturally occur are however exposed to fluctuations in light, nutrients, temperature, as well as changes in species composition due to migrations and water currents. In order to achieve higher comparability with natural environments, further investigations should focus on the effects of temperature variations and other abiotic fluctuations and their effects on competition between these two diatoms. Other species combinations and more complex communities could also be investigated to understand how general the current findings are. The ability to predict where inflexion points of species interactions lie across gradients and when multiple environmental stressors interact will bring the scientific community closer to understanding nonlinear ecosystem responses and help to potentially find strategies to mitigate changes that are predicted to occur under future environmental scenarios.

## CONFLICT OF INTEREST

None declared.

## AUTHOR CONTRIBUTIONS

**Philipp Siegel:** Conceptualization (lead); Data curation (lead); Formal analysis (lead); Investigation (lead); Methodology (lead); Project administration (lead); Validation (lead); Visualization (lead); Writing‐original draft (lead); Writing‐review & editing (lead). **Kirralee G. Baker:** Data curation (supporting); Formal analysis (supporting); Investigation (supporting); Methodology (supporting); Project administration (supporting); Validation (supporting); Writing‐original draft (supporting); Writing‐review & editing (supporting). **Etienne Low‐Décarie:** Conceptualization (supporting); Data curation (supporting); Formal analysis (supporting); Funding acquisition (lead); Supervision (lead); Validation (supporting); Visualization (supporting); Writing‐original draft (supporting); Writing‐review & editing (supporting). **Richard J. Geider:** Conceptualization (supporting); Data curation (supporting); Formal analysis (supporting); Funding acquisition (supporting); Investigation (supporting); Methodology (supporting); Supervision (supporting); Validation (supporting); Visualization (supporting); Writing‐original draft (supporting); Writing‐review & editing (supporting).

## Supporting information

Supplementary Material

## Data Availability

Collected data and R script used for the analysis of the publication can be downloaded via the publicly accessible repository “Knowledge Network for Biocomplexity” under https://knb.ecoinformatics.org/view/urn:uuid:11f6dc8a‐91cc‐4c9d‐8cf0‐00366b3b0374.
