# Supplementary material for: High predictability of direct competition between marine diatoms under different temperatures and nutrient states
Source: Ecol Evol. 2020 Jun 3;10(14):7276–90. doi: 10.1002/ece3.6453 (PMC7391539; doi:10.1002/ece3.6453)
Supplement: Supplementary file 1 — Supplementary Material [file ECE3-10-7276-s001.docx]

# Supplementary Tables

Supplementary Table S1: A list of equations that can be used to describe thermal performance across temperatures from Low-Décarie et al. (2017). The experimental growth rates and carrying capacity in this study were fitted to Equations 4 – 15 (shaded in green) with the R package “temperatureresponse”. Equations 1 – 3 (shaded in blue) can only be used to cover a sub- or supra-optimal range of temperatures, not entire temperature range, and were therefore not utilised in this study.

| **Formula** | **Equ.** | **Number of Parameters** | **Reference** |
| --- | --- | --- | --- |
| $k(T)=a\cdot exp\left( \frac{-E_{A}}{R\cdot T} \right)$ | 1 | 2 | (Raven and Geider, 1988) |
| $k\left( T \right)=k(T_{ref})\cdot exp\left\lceil-\frac{E_{A}}{R}\left( \frac{1}{T}-\frac{1}{T_{ref}} \right) \right\rceil$ | 2 | 2 | e.g.(Geider et al., 1997; Li et al., 1984) |
| $\mu(T)= \mu_{0}\cdot exp\left( k\cdot T \right)$ | 3 | 2 | (Eppley, 1972) |
| $Rate=a\cdot exp\left( \frac{-b}{R\cdot T} \right)-c\cdot exp\left( \frac{-d}{R\cdot T} \right)$ | 4 | 4 | (Li and Dickie, 1987) citing(Hinshelwood, 1947) |
| $Rate=\frac{a\cdot T\cdot exp\left( \frac{-b}{R\cdot T} \right)}{1+exp\left( \frac{-c}{R} \right)\cdot exp\left( \frac{-d}{R\cdot T} \right)}$ | 5 | 4 | (Li and Dickie, 1987) citing  (Johnson et al., 1942) |
| $Rate= \frac{a\cdot\left( \frac{T}{298.15} \right)\cdot exp\left( \frac{b}{R}\cdot\left( \frac{1}{298.15}-\frac{1}{T} \right) \right)}{1+exp\left[ \frac{c}{R}\cdot\left( \frac{1}{d}-\frac{1}{T} \right) \right]+exp\left[ \frac{e}{R}\left( \frac{1}{f}-\frac{1}{T} \right) \right]}$ | 6 | 6 | (Heitzer et al., 1991) |
| $Rate= \frac{a\cdot\left( \frac{T}{293.15} \right)\cdot exp\left( \frac{b}{R}\cdot\left( \frac{1}{293.15}-\frac{1}{T} \right) \right)}{1+exp\left[ \frac{c}{R}\cdot\left( \frac{1}{d}-\frac{1}{T} \right) \right]}$ | 7 | 4 | (Montagnes et al., 2008) citing (Schoolfield et al., 1981) |
| $Rate=a\cdot exp\left[ -0.5\cdot\left( \frac{\left[ T-T_{ref} \right]}{b} \right)^{2} \right]$ | 8 | 3 | (Li and Dickie, 1987) citing (Stoermer and Ladewski, 1976) |
| $Rate= a\cdot exp\left[ -0.5\cdot\left( \frac{abs\left\lceil T-T_{ref} \right\rceil}{b} \right)^{c} \right]$ | 9 | 4 | (Montagnes et al., 2008) |
| $Rate= a\cdot exp\left( c\cdot T \right)\left[ 1-\left( \frac{T-T_{ref}}{b} \right)^{2} \right]$ | 10 | 4 | (Thomas et al., 2012) |
| $Rate=a+b\cdot T+c\cdot T^{2}$ | 11 | 3 | (Montagnes et al., 2008) |
| $Rate= \frac{1}{1+\left( a+b\cdot T+c\cdot T^{2} \right)}$ | 12 | 3 | (Montagnes et al., 2008) citing (Flinn, 1991) |
| $Rate=\left[ a\cdot\left( T-T_{min} \right) \right]^{2}\cdot\left[ 1-exp\left( b\cdot\left( T-T_{max} \right) \right) \right]^{2}$ | 13 | 4 | (Ratkowsky et al., 1983) |
| $Rate= a\cdot\left\{ 1-exp\left[ -b\cdot\left( T-T_{min} \right) \right] \right\}\cdot\left\{ 1-exp\left[ -c\cdot\left( T_{max}-T \right) \right] \right\}$ | 14 | 5 | (Kamykowski, 1986) |
| $Rate= R_{max}\cdot\left\{ sin\left[ \pi\cdot\left( \frac{T-T_{min}}{T_{max}-T_{min}} \right)^{a} \right] \right\}^{b}$ | 15 | 5 | (Boatman et al., 2017) |

**Supplementary Table S2:** Non-linear model outputs of single replicate TPC growth models for *P. tricornutum* (n = 4) and *T. pseudonana* (n = 4, of which replicate 2 was removed for final analysis) in the high N scenario.

| **Species** | **Equ.** | **replicate** | **term** | **estimate** | **std.err.** | **statistic** | **p.value** |
| --- | --- | --- | --- | --- | --- | --- | --- |
| *P. tricornutum* | Equ. 13 | 1 | a | 0.0230 | 0.005 | 4.463617 | 0.0021 |
|  |  | 1 | b | 0.3550 | 0.154 | 2.309595 | 0.0497 |
|  |  | 1 | T_opt_ | 22.27 |  |  |  |
|  |  | 1 | µ_max_ | 0.81 |  |  |  |
|  |  | 1 | AIC | -32.23 |  |  |  |
|  |  | 1 | BIC | -29.80 |  |  |  |
|  |  | 1 | pseudoR^2^ | 0.893 |  |  |  |
|  |  | 1 | T_50min_ | 8.15 |  |  |  |
|  |  | 1 | T_50max_ | 27.56 |  |  |  |
| *P. tricornutum* | Equ. 13 | 2 | a | 0.0233 | 0.002 | 10.49028 | 2.4E-06 |
|  |  | 2 | b | 0.360 | 0.048 | 7.499019 | 3.7E-05 |
|  |  | 2 | T_opt_ | 22.64 |  |  |  |
|  |  | 2 | µ_max_ | 0.82 |  |  |  |
|  |  | 2 | AIC | -51.44 |  |  |  |
|  |  | 2 | BIC | -48.62 |  |  |  |
|  |  | 2 | pseudoR^2^ | 0.980 |  |  |  |
|  |  | 2 | T_50min_ | 8.66 |  |  |  |
|  |  | 2 | T_50max_ | 27.86 |  |  |  |
| *P. tricornutum* | Equ. 13 | 3 | a | 0.0212 | 0.001 | 17.19604 | 3.43E-08 |
|  |  | 3 | b | 0.4520 | 0.038 | 12.00063 | 7.7E-07 |
|  |  | 3 | T_opt_ | 23.18 |  |  |  |
|  |  | 3 | µ_max_ | 0.85 |  |  |  |
|  |  | 3 | AIC | -60.78 |  |  |  |
|  |  | 3 | BIC | -57.95 |  |  |  |
|  |  | 3 | pseudoR^2^ | 0.99 |  |  |  |
|  |  | 3 | T_50min_ | 8.35 |  |  |  |
|  |  | 3 | T_50max_ | 27.89 |  |  |  |
| *P. tricornutum* | Equ. 13 | 4 | a | 0.0222 | 0.002 | 11.59617 | 1.03E-06 |
|  |  | 4 | b | 0.4069 | 0.051 | 7.933699 | 2.37E-05 |
|  |  | 4 | T_opt_ | 23.03 |  |  |  |
|  |  | 4 | µ_max_ | 0.84 |  |  |  |
|  |  | 4 | AIC | -51.84 |  |  |  |
|  |  | 4 | BIC | -49.01 |  |  |  |
|  |  | 4 | pseudoR^2^ | 0.98 |  |  |  |
|  |  | 4 | T_50min_ | 8.65 |  |  |  |
|  |  | 4 | T_50max_ | 27.97 |  |  |  |
|  |  |  |  |  |  |  |  |
| *T. pseudonana* | Equ. 11 | 1 | a | -1.4794 | 0.3294 | -4.49186 | 0.0009 |
|  |  | 1 | b | 0.1702 | 0.0282 | 6.03787 | 8.45E-05 |
|  |  | 1 | c | -0.0029 | 0.0006 | -4.9962 | 0.0004 |
|  |  | 1 | T_opt_ | 29.73 |  |  |  |
|  |  | 1 | µ_max_ | 1.05 |  |  |  |
|  |  | 1 | AIC | -26.28 |  |  |  |
|  |  | 1 | BIC | -23.73 |  |  |  |
|  |  | 1 | pseudoR^2^ | 0.90 |  |  |  |
|  |  | 1 | T_50min_ | 16.18 |  |  |  |
|  |  | 1 | T_50max_ | 43.29 |  |  |  |
| *T. pseudonana* | Equ. 11 | 2 | a | -0.5602 | 0.4033 | -1.38885 | 0.1950 |
|  |  | 2 | b | 0.0954 | 0.0331 | 2.88061 | 0.0164 |
|  |  | 2 | c | -0.0014 | 0.0007 | -2.15476 | 0.0566 |
|  |  | 2 | T_opt_ | 33.96 |  |  |  |
|  |  | 2 | µ_max_ | 1.06 |  |  |  |
|  |  | 2 | AIC | -25.66 |  |  |  |
|  |  | 2 | BIC | -23.40 |  |  |  |
|  |  | 2 | pseudoR^2^ | 0.83 |  |  |  |
|  |  | 2 | T_50min_ | 14.54 |  |  |  |
|  |  | 2 | T_50max_ | 53.38 |  |  |  |
| *T. pseudonana* | Equ. 11 | 3 | a | -1.7995 | 0.4051 | -4.44268 | 0.0009 |
|  |  | 3 | b | 0.2122 | 0.0347 | 6.11901 | 7.53E-05 |
|  |  | 3 | c | -0.0040 | 0.0007 | -5.64442 | 0.0002 |
|  |  | 3 | T_opt_ | 26.67 |  |  |  |
|  |  | 3 | µ_max_ | 1.03 |  |  |  |
|  |  | 3 | AIC | -20.49 |  |  |  |
|  |  | 3 | BIC | -17.94 |  |  |  |
|  |  | 3 | pseudoR^2^ | 0.81 |  |  |  |
|  |  | 3 | T_50min_ | 15.29 |  |  |  |
|  |  | 3 | T_50max_ | 38.05 |  |  |  |
| *T. pseudonana* | Equ. 11 | 4 | a | -1.5748 | 0.1220 | -12.9132 | 8.63E-09 |
|  |  | 4 | b | 0.1843 | 0.0114 | 16.10451 | 5.73E-10 |
|  |  | 4 | c | -0.003 | 0.0002 | -12.7258 | 1.03E-08 |
|  |  | 4 | T_opt_ | 29.09 |  |  |  |
|  |  | 4 | µ_max_ | 1.11 |  |  |  |
|  |  | 4 | AIC | -47.50 |  |  |  |
|  |  | 4 | BIC | -44.41 |  |  |  |
|  |  | 4 | pseudoR^2^ | 0.98 |  |  |  |
|  |  | 4 | T_50min_ | 15.88 |  |  |  |
|  |  | 4 | T_50max_ | 42.31 |  |  |  |

**Supplementary Table S3:** Non-linear model outputs of single replicate TPC growth models for *P. tricornutum* (n = 2) and *T. pseudonana* (n = 2) in the low N scenario.

| **Species** | **Equ.** | **replicate** | **term** | **estimate** | **std.err** | **statistic** | **p.value** |
| --- | --- | --- | --- | --- | --- | --- | --- |
| *P. tricornutum* | Equ.13 | 1 | a | 0.0320 | 0.0020 | 16.17818 | 2.14E-07 |
|  |  | 1 | b | 0.2454 | 0.0299 | 8.200413 | 3.65E-05 |
|  |  | 1 | T_opt_ | 22.04 |  |  |  |
|  |  | 1 | µ_max_ | 0.96 |  |  |  |
|  |  | 1 | AIC | -65.32 |  |  |  |
|  |  | 1 | BIC | -62.89 |  |  |  |
|  |  | 1 | pseudoR^2^ | 1.00 |  |  |  |
|  |  | 1 | T_50min_ | 9.54 |  |  |  |
|  |  | 1 | T_50max_ | 28.03 |  |  |  |
| *P. tricornutum* | Equ.13 | 2 | a | 0.0256 | 0.0072 | 3.557295 | 0.0074 |
|  |  | 2 | b | 0.3012 | 0.1663 | 1.811701 | 0.1076 |
|  |  | 2 | T_opt_ | 22.10 |  |  |  |
|  |  | 2 | µ_max_ | 0.94 |  |  |  |
|  |  | 2 | AIC | -28.35 |  |  |  |
|  |  | 2 | BIC | -25.92 |  |  |  |
|  |  | 2 | pseudoR^2^ | 0.87 |  |  |  |
|  |  | 2 | T_50min_ | 7.97 |  |  |  |
|  |  | 2 | T_50max_ | 27.87 |  |  |  |
|  |  |  |  |  |  |  |  |
| *T. pseudonana* | Equ.11 | 1 | a | -0.8615 | 0.1434 | -6.00656 | 4.4E-05 |
|  |  | 1 | b | 0.12812 | 0.0135 | 9.487492 | 3.3E-07 |
|  |  | 1 | c | -0.0024 | 0.0003 | -7.9881 | 2.27E-06 |
|  |  | 1 | T_opt_ | 27.01 |  |  |  |
|  |  | 1 | µ_max_ | 0.87 |  |  |  |
|  |  | 1 | AIC | -45.36 |  |  |  |
|  |  | 1 | BIC | -42.27 |  |  |  |
|  |  | 1 | pseudoR^2^ | 0.93 |  |  |  |
|  |  | 1 | T_50min_ | 13.48 |  |  |  |
|  |  | 1 | T_50max_ | 40.55 |  |  |  |
| *T. pseudonana* | Equ.11 | 2 | a | -0.8858 | 0.1614 | -5.48943 | 0.0001 |
|  |  | 2 | b | 0.1329 | 0.0145 | 9.158956 | 9.17E-07 |
|  |  | 2 | c | -0.0025 | 0.0003 | -8.10409 | 3.29E-06 |
|  |  | 2 | T_opt_ | 26.60 |  |  |  |
|  |  | 2 | µ_max_ | 0.88 |  |  |  |
|  |  | 2 | AIC | -46.76 |  |  |  |
|  |  | 2 | BIC | -43.92 |  |  |  |
|  |  | 2 | pseudoR^2^ | 0.92 |  |  |  |
|  |  | 2 | T_50min_ | 13.32 |  |  |  |
|  |  | 2 | T_50max_ | 39.89 |  |  |  |

**Supplementary Table S4:** Non-linear model outputs of single replicate TPC carrying capacity models for P*. tricornutum* (n = 2) and *T. pseudonana* (n = 2) in the low N scenario.

| **Species** | **Equ.** | **replicate** | **term** | **estimate** | **std.err.** | **statistic** | **p.value** |
| --- | --- | --- | --- | --- | --- | --- | --- |
| *P. tricornutum* | Equ.13 | 1 | a | 0.0398 | 0.0145 | 2.750692 | 0.0225 |
|  |  | 1 | b | 0.1966 | 0.0855 | 2.298777 | 0.0471 |
|  |  | 1 | T_opt_ | 19.96 |  |  |  |
|  |  | 1 | µ_max_ | 0.73 |  |  |  |
|  |  | 1 | AIC | -26.93 |  |  |  |
|  |  | 1 | BIC | -24.11 |  |  |  |
|  |  | 1 | pseudoR^2^ | 0.91 |  |  |  |
|  |  | 1 | T_50min_ | 9.78 |  |  |  |
|  |  | 1 | T_50max_ | 25.77 |  |  |  |
| *P. tricornutum* | Equ.13 | 2 | a | 0.0474 | 0.0118 | 4.022561 | 0.0030 |
|  |  | 2 | b | 0.1925 | 0.0606 | 3.173647 | 0.0113 |
|  |  | 2 | T_opt_ | 19.97 |  |  |  |
|  |  | 2 | µ_max_ | 0.77 |  |  |  |
|  |  | 2 | AIC | -33.83 |  |  |  |
|  |  | 2 | BIC | -31.00 |  |  |  |
|  |  | 2 | AICc | -25.26 |  |  |  |
|  |  | 2 | T_50min_ | 10.75 |  |  |  |
|  |  | 2 | T_50max_ | 25.47 |  |  |  |
|  |  |  |  |  |  |  |  |
| *T. pseudonana* | Equ.11 | 1 | a | -1.1851 | 0.1654 | -7.1666 | 4.81E-06 |
|  |  | 1 | b | 0.1438 | 0.0164 | 8.765961 | 4.65E-07 |
|  |  | 1 | c | -0.0028 | 0.0004 | -7.50346 | 2.86E-06 |
|  |  | 1 | T_opt_ | 25.57 |  |  |  |
|  |  | 1 | µ_max_ | 0.65 |  |  |  |
|  |  | 1 | AIC | -34.05 |  |  |  |
|  |  | 1 | BIC | -30.72 |  |  |  |
|  |  | 1 | pseudoR^2^ | 0.90 |  |  |  |
|  |  | 1 | T_50min_ | 14.79 |  |  |  |
|  |  | 1 | T_50max_ | 36.35 |  |  |  |
| *T. pseudonana* | Equ.11 | 2 | a | -1.26407 | 0.1382 | -9.14787 | 2.78E-07 |
|  |  | 2 | b | 0.1556 | 0.0137 | 11.34796 | 1.91E-08 |
|  |  | 2 | c | -0.0031 | 0.0003 | -9.89238 | 1.07E-07 |
|  |  | 2 | T_opt_ | 25.11 |  |  |  |
|  |  | 2 | µ_max_ | 0.69 |  |  |  |
|  |  | 2 | AIC | -40.15 |  |  |  |
|  |  | 2 | BIC | -36.82 |  |  |  |
|  |  | 2 | pseudoR^2^ | 0.93 |  |  |  |
|  |  | 2 | T_50min_ | 14.56 |  |  |  |
|  |  | 2 | T_50max_ | 35.65 |  |  |  |

**Inflexion Point comparison**

**Supplementary Table S5:** One-way ANOVA output for comparison of competition inflexion point temperature between unacclimated and acclimated competition replicates in the high N scenario.


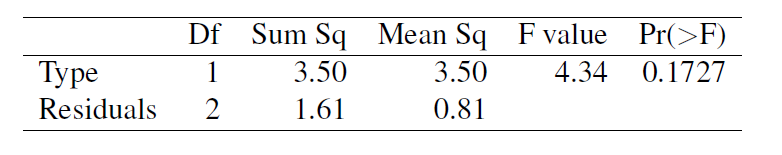


**High N and low N growth rate MANOVA fits**

**Supplementary Table S6:** Two-way MANOVA output table for comparison between growth rate thermal performance curve (TPC) fits of *P. tricornutum* and *T. pseudonana*. Dependent variables that were compared were µ_max_, CT_50min_, CT_50max_, and T_opt_. Individual ANOVA test statistics wrapped within this MANOVA are found in Supplementary Tables S7 – S10.


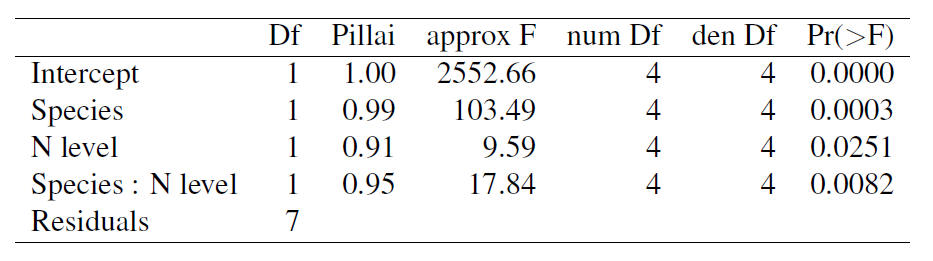


**Supplementary Table S7:** Two-way Individual ANOVA output table from MANOVA for CT_50min_.


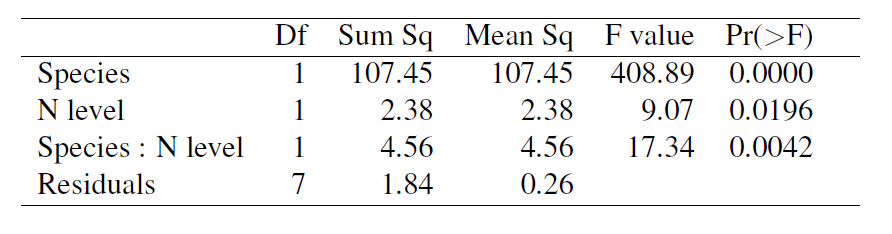


**Supplementary Table S8:** Two-way Individual ANOVA output table from MANOVA for T_opt_.


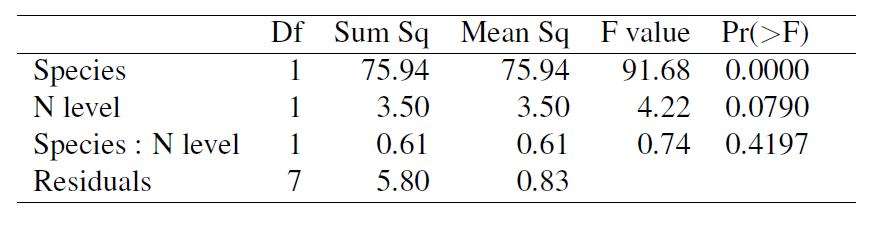


**Supplementary Table S9:** Two-way Individual ANOVA output table from MANOVA for CT_50max_.


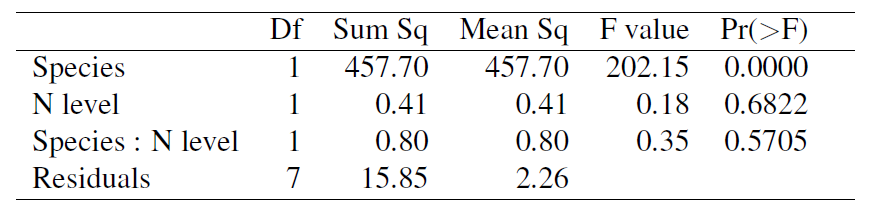


**Supplementary Table S10:** Two-way Individual ANOVA output table from MANOVA for µ_max_.


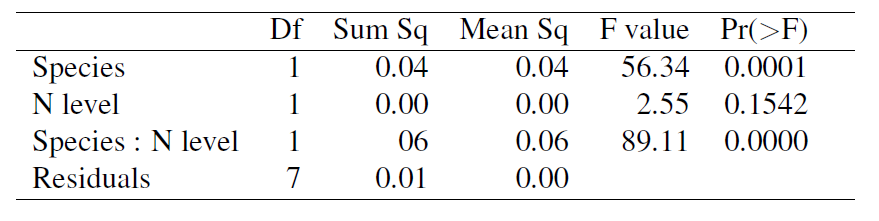


**Low N carrying capacity ANOVA fits**

**Supplementary Table S11:** One-way ANOVA output table of CT_50min_ from K model fits.


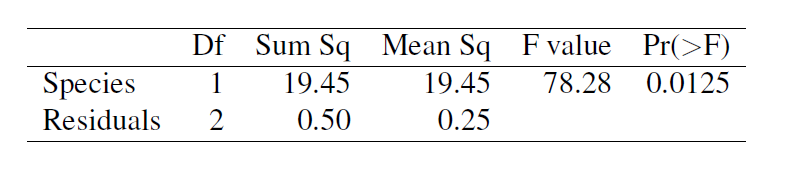


**Supplementary Table S12:** One-way ANOVA output table of T_opt_ from K model fits.


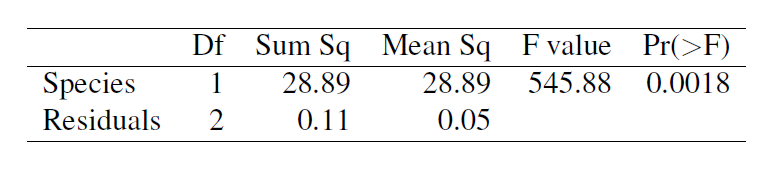


**Supplementary Table S13:** One-way ANOVA output table of CT_50max_ from K model fits.


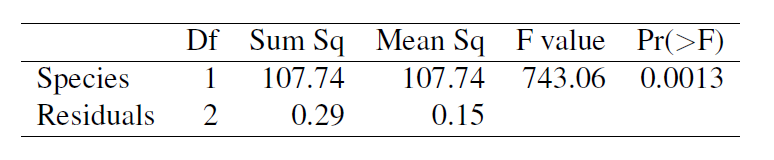


**Supplementary Table S14:** One-way ANOVA output table of K_max_ from K model fits.


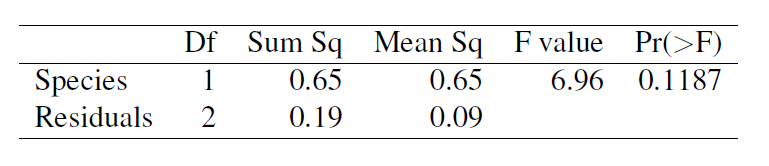


**Observed Inflexion Point Comparison**

**Supplementary Table S15:** One-way ANOVA output table for differences between high N and low N inflexion points.


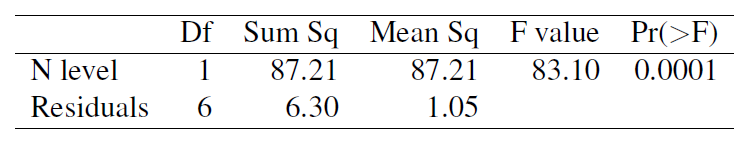


# Supplementary Figures


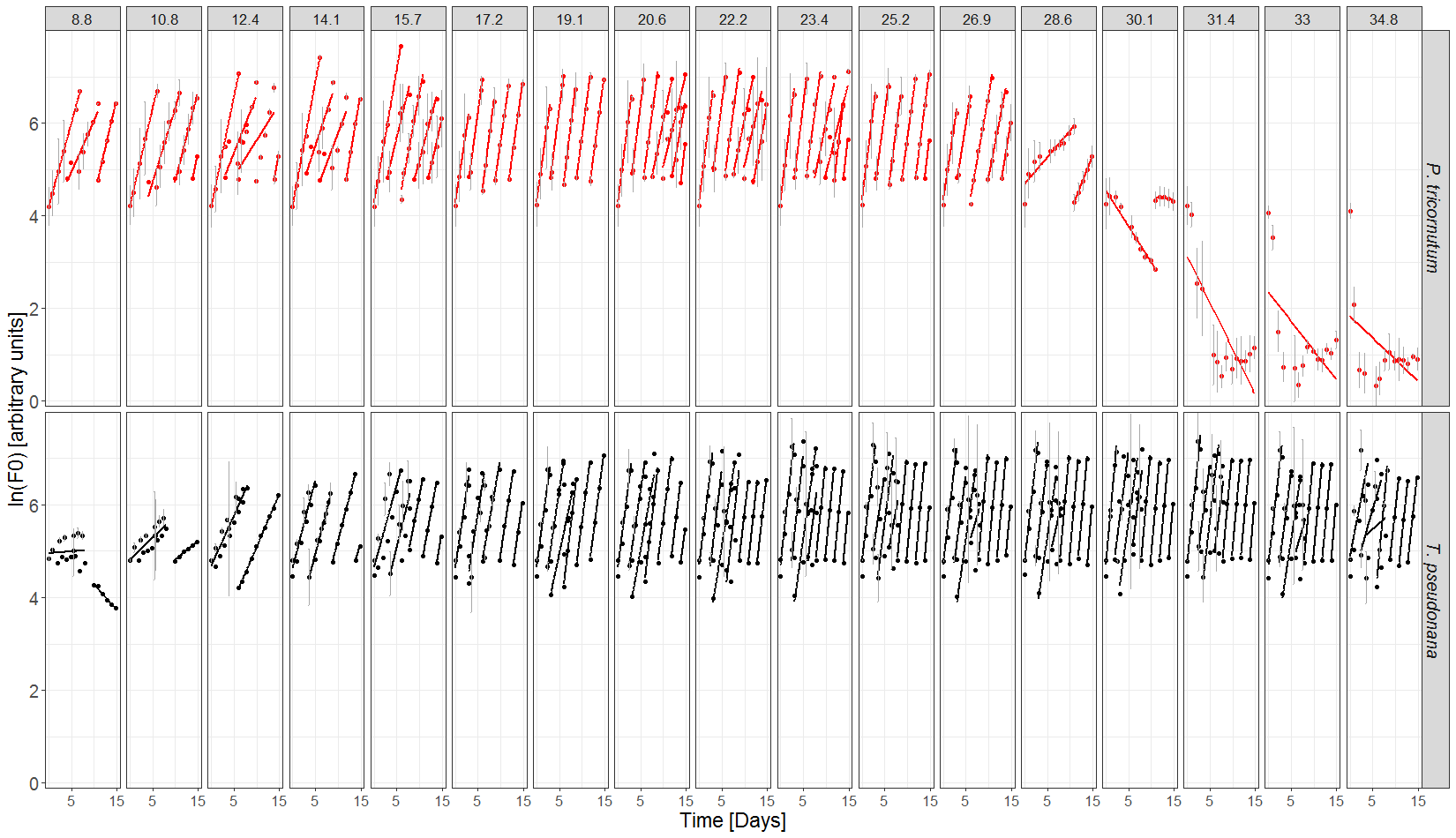
Supplementary Figure S1: Average ln(*F0*) of exponentially growing *P. tricornutum* (n = 4) (red) and *T. pseudonana* (n = 3) (black) in the high N medium measured at the indicated assay temperatures across the temperature gradient. Individual slopes represent single dilution phases. Grey error bars denote 1 standard deviation.


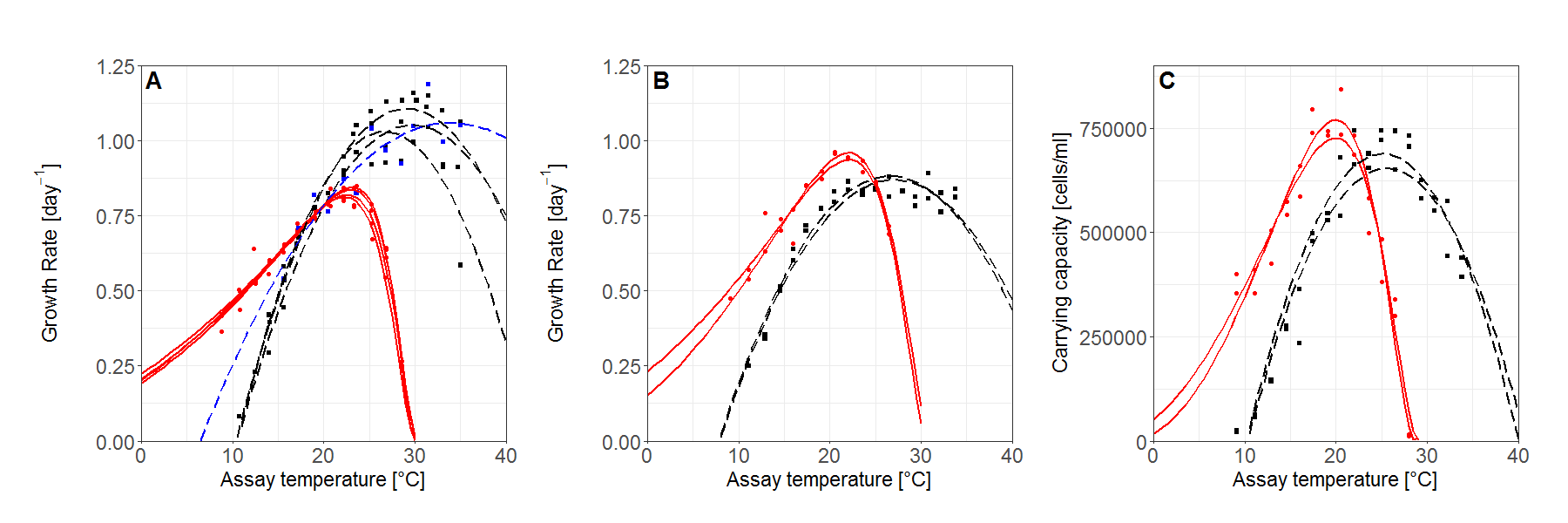


**Supplementary Figure S2:** (A) Single TPC model fits for the two diatom species *P. tricornutum* in red (n=4) and *T. pseudonana* in black (n=4) from which average growth models for Figure 2A were calculated. The single *T. pseudonana* replicate that was omitted in the final analysis is plotted in blue. (B) Single TPC model fits for the two diatom species *P. tricornutum* in red (n=2) and *T. pseudonana* in black (n=2) from which average growth models for Figure 2B were calculated. (C) Single TPC model fits for the two diatom species *P. tricornutum* in red (n=2) and *T. pseudonana* in black (n=2) from which average carrying capacity models across temperatures for Figure 4A were calculated. Single non-linear model parameters for these model fits can be found in Supplementary Tables S2 – S4.


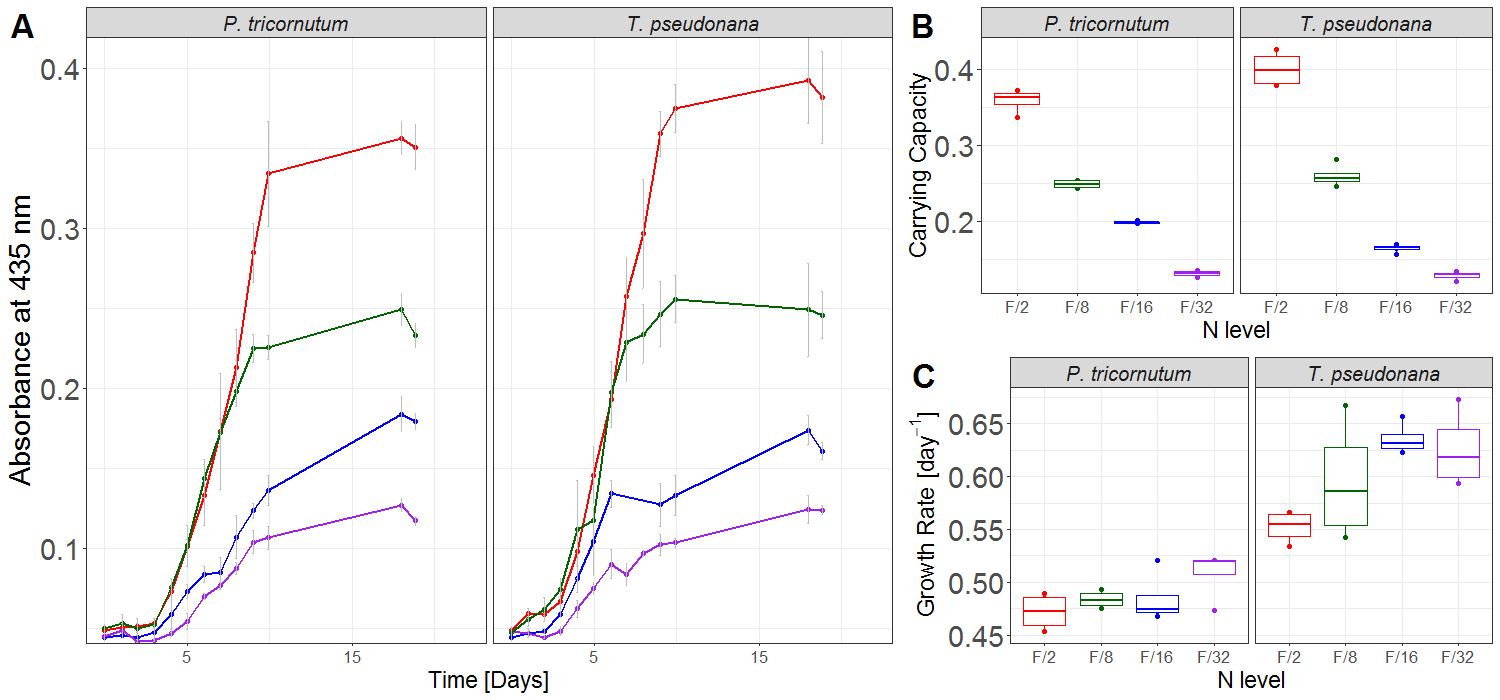


Supplementary Figure S3: (A) Growth progression over time for *P. tricornutum* and *T. pseudonana* grown in F/2 medium with different nitrate concentrations (882µM (red), 220.5 µM (green), 110.25 µM (blue), 55.13 µM (purple)). (B) Dependence of carrying capacity (K) on nitrogen concentration in the growth medium. (C) Dependence of exponential growth rate r on starting nitrogen concentration in the growth medium.


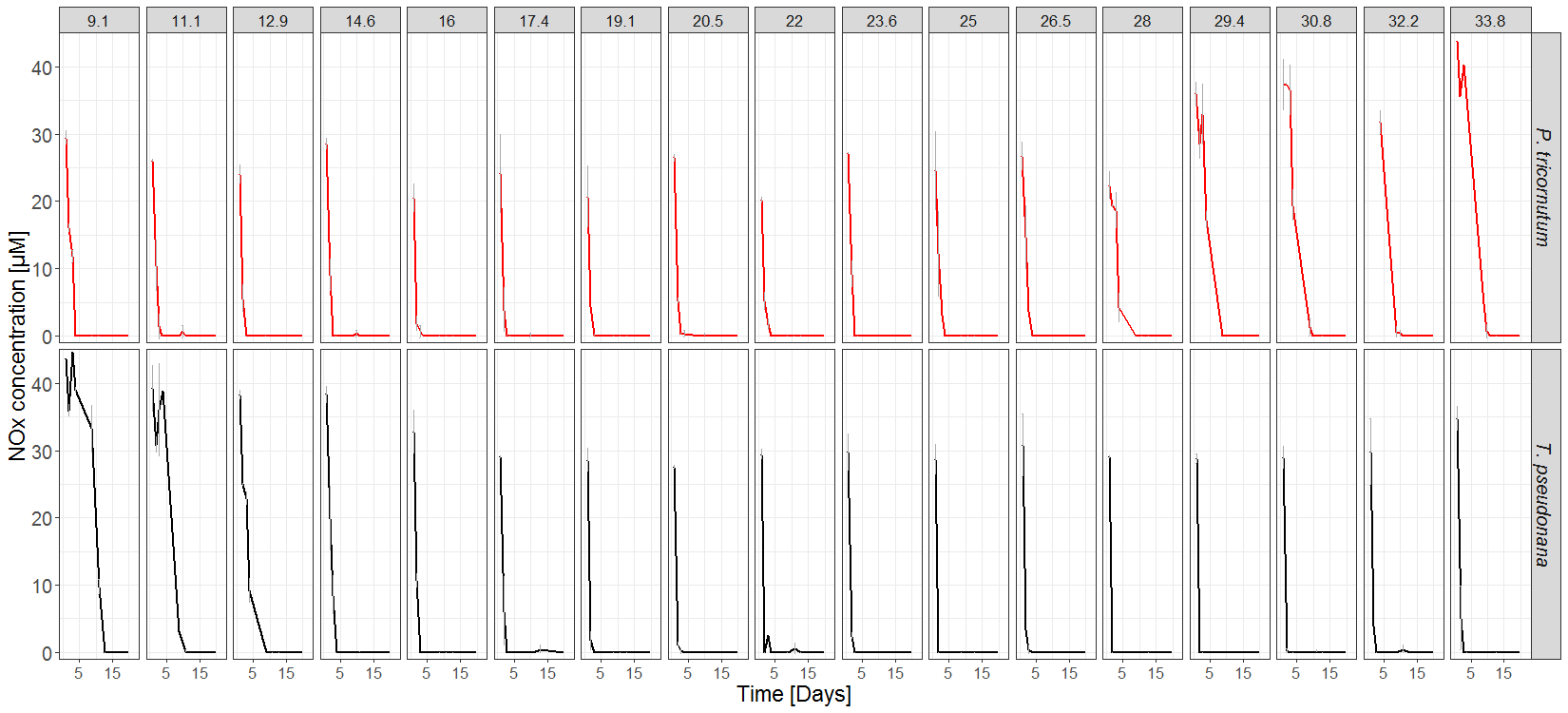


Supplementary Figure S4: Average (n = 2) nitrate plus nitrite concentrations in monocultures across all assay temperatures in the low N medium over time for *P. tricornutum* (red) and *T. pseudonana* (black)*.* Grey error bars denote standard deviation from two replicates.


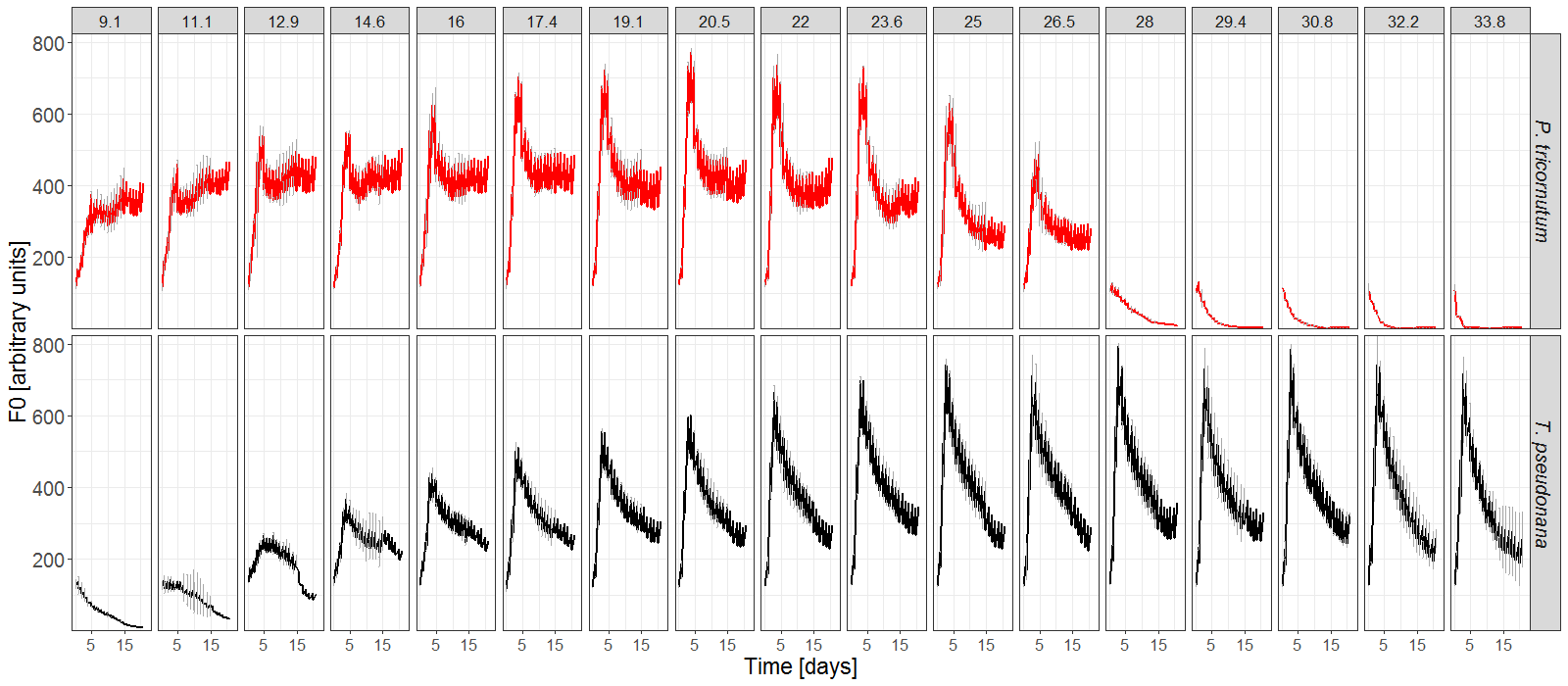


Supplementary Figure S5: Average (n = 2) *F_0_* fluorescence in low N monocultures across all assay temperatures over time for *P. tricornutum* (red) and *T. pseudonana* (black)*.* Grey error bars denote standard deviation from two replicates.

**
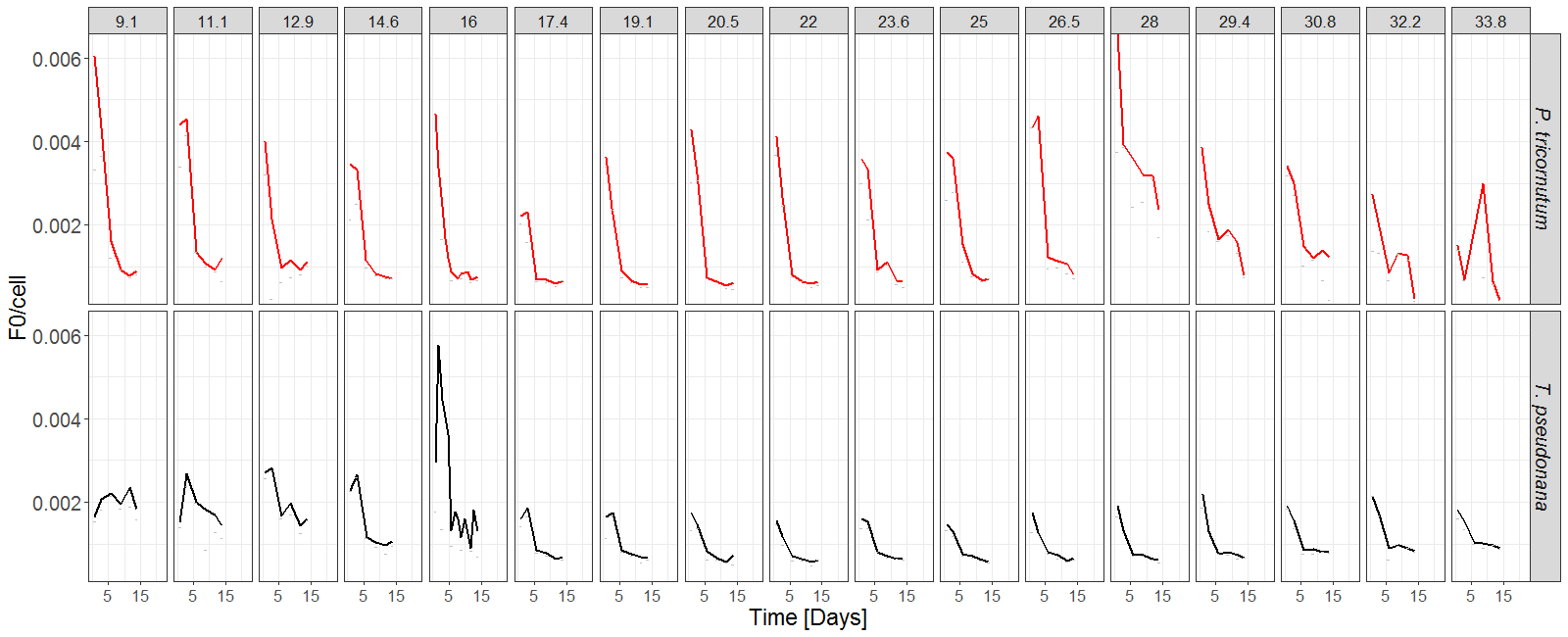
**

**Supplementary Figure S6:** Average (n = 2) *F_0_* measured by FRRf per cell across all assay temperatures for *P. tricornutum* (red) and *T. pseudonana* (black) in low N medium. Flow cytometry estimates of cell abundance were taken for monocultures in the first 14 days of the experiment before mixing cultures together to start competition experiments. Grey error bars denote standard deviation from two replicates.


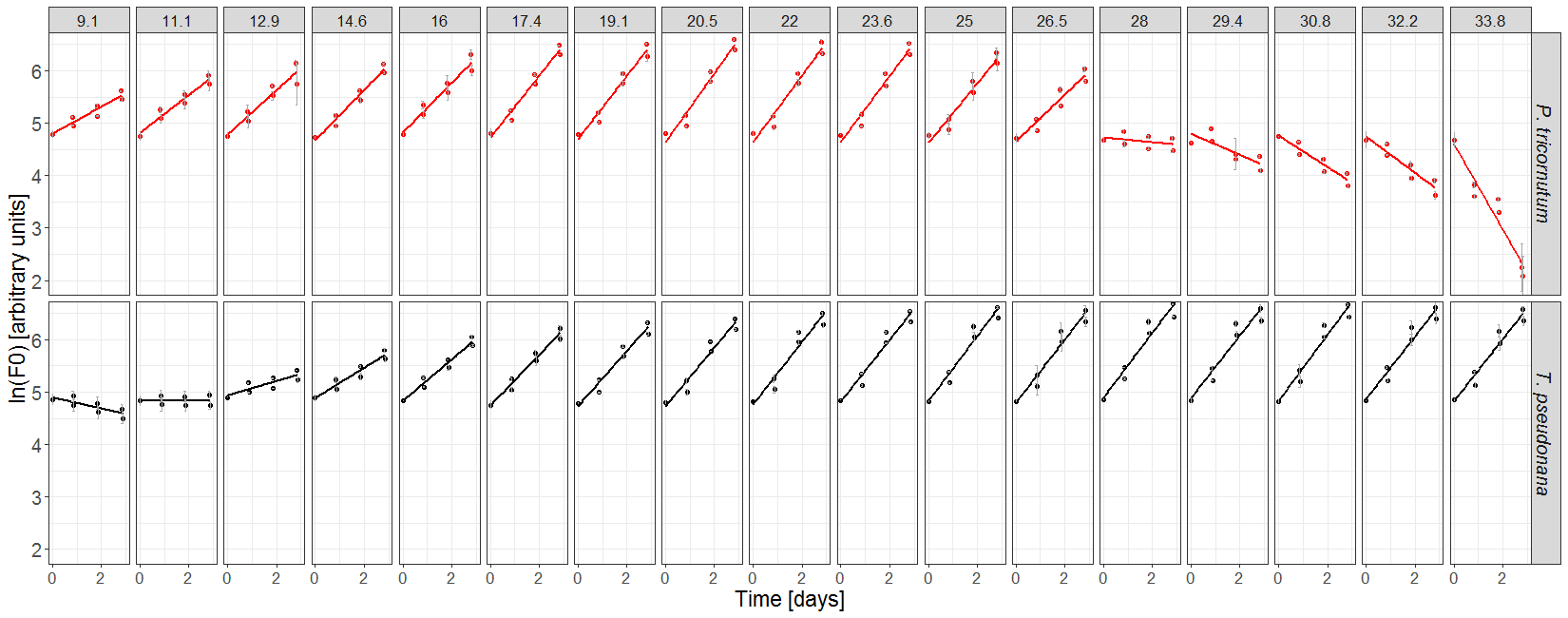


Supplementary Figure S7: Mean slopes of ln(*F_0_*) across temperatures for *P. tricornutum* (n = 2) and *T. pseudonana* (n = 2) during the first four days of the low N scenario. Single replicate slopes were used to calculate the acute exponential growth response across temperatures, to which a daily growth rate of 0.22 d^-1^ was added to account for the daily dilution rates. Single growth replicate rates can be seen in Fig. 2B as symbols. Grey error bars denote 1 standard deviation.


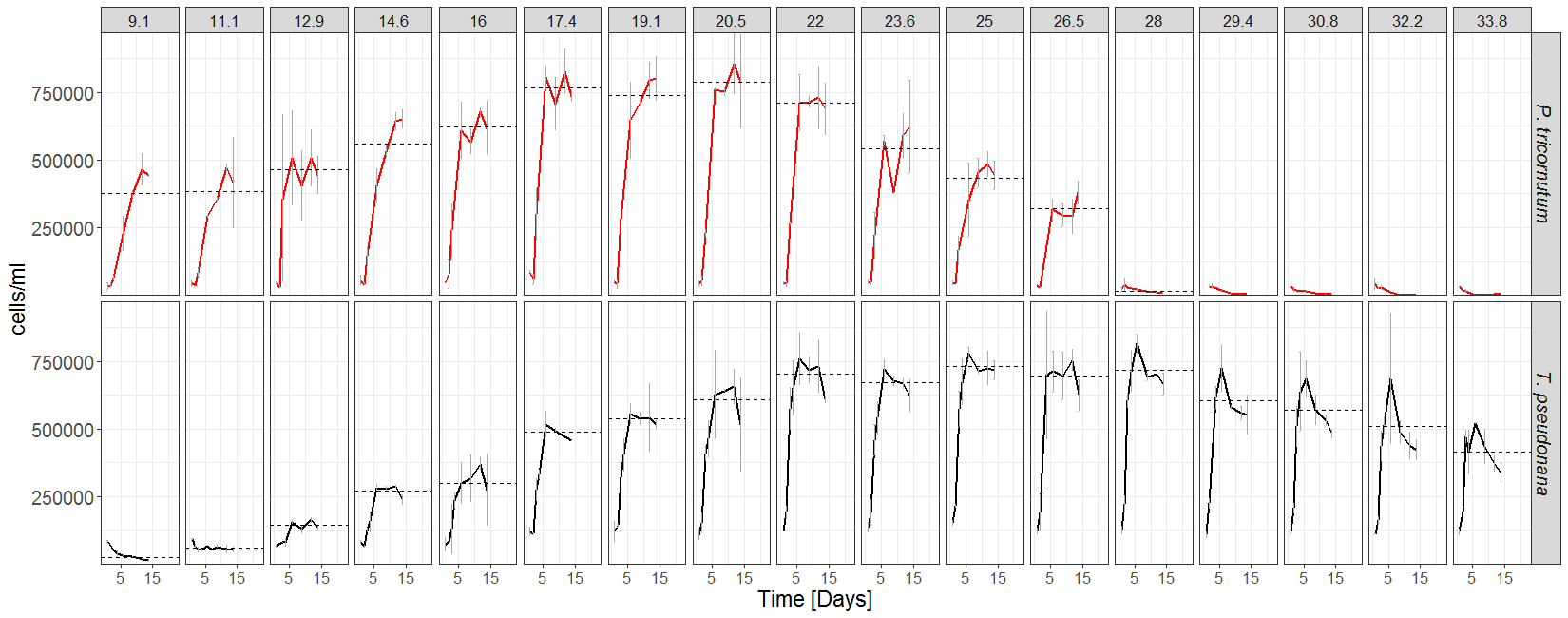


Supplementary Figure S8: Average (n = 2) cells per ml across all assay temperatures for *P. tricornutum* and *T. pseudonana* in low N medium. Flow cytometry counts were taken for monocultures in the first 14 days of the experiment before mixing cultures together to start competition experiments. Grey error bars denote standard deviation from two replicates. Dashed line in each facet panel is the average carrying capacity for each monoculture growth duplicate from day 5 onwards across assay temperatures.


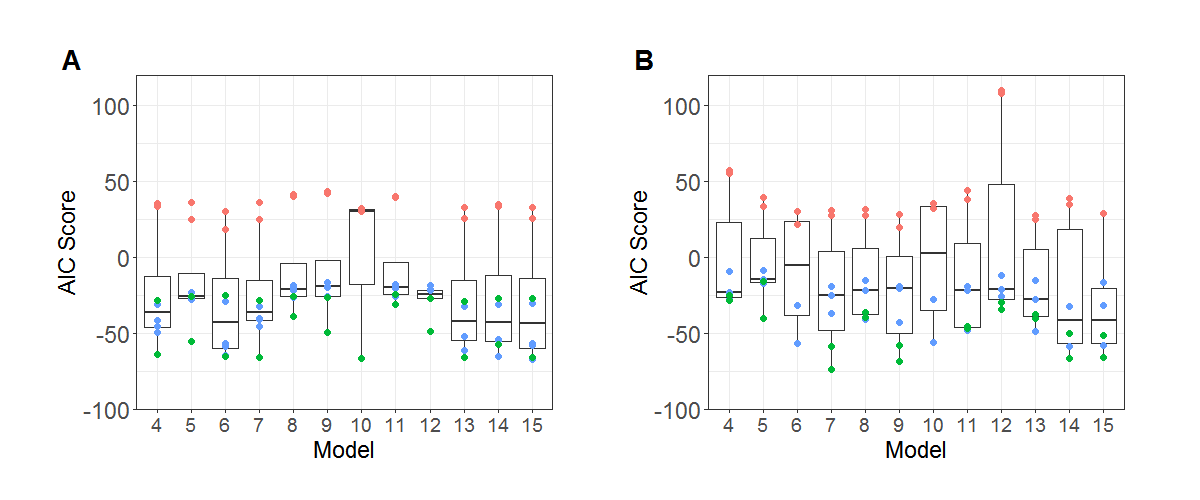


Supplementary Figure S9: All AIC values across models from the models fits of high N growth rates (blue symbols), low N growth rates (green symbols), and low N carrying capacities (red symbols) across temperatures for (A) *P. tricornutum* and (B) *T. pseudonana*. AIC scores for *T. pseudonana* replicate 2 are excluded as it was not taken into consideration for the final data analysis.


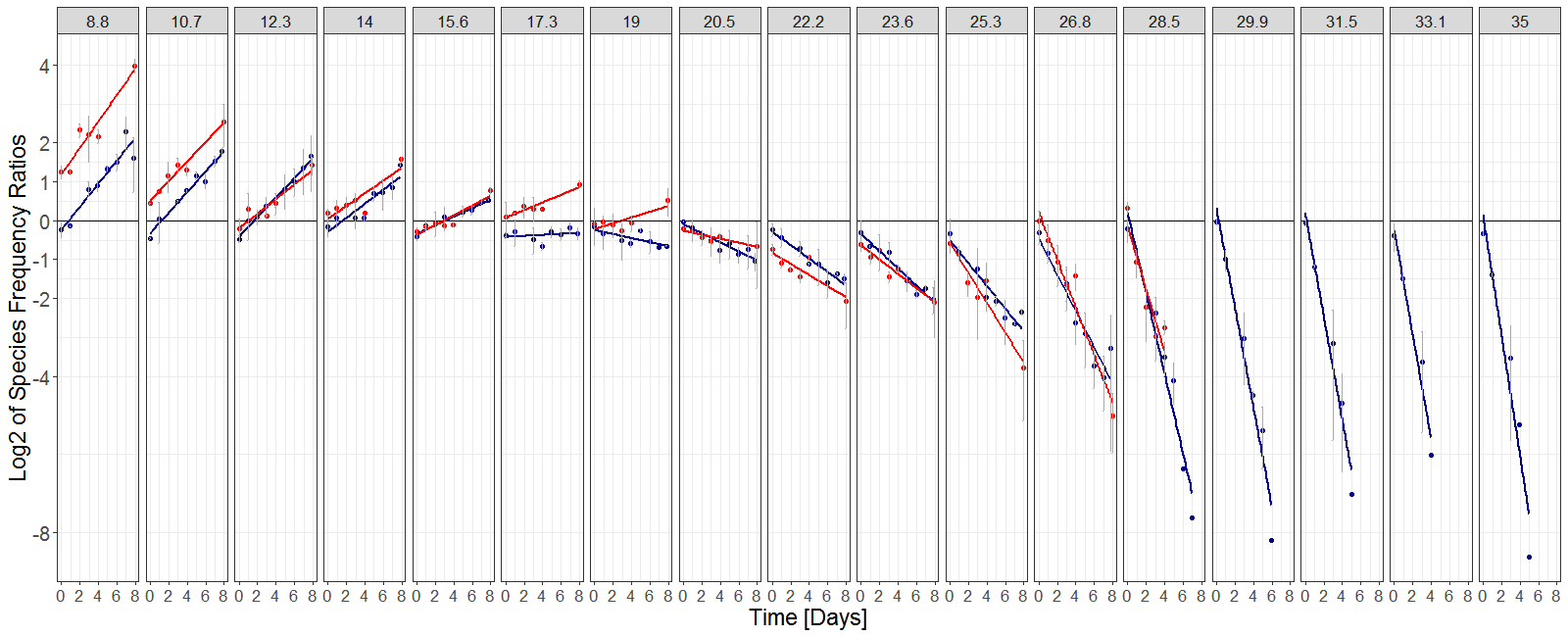


Supplementary Figure S10: Log_2_ over time of *P. tricornutum*-to-*T. pseudonana* cell abundance ratios in high N mixed cultures. Blue lines are the first 2 competition replicates that were started directly at the beginning of the experiment. Red lines are the temperature acclimated competition replicates that were started after the first competition was completed.


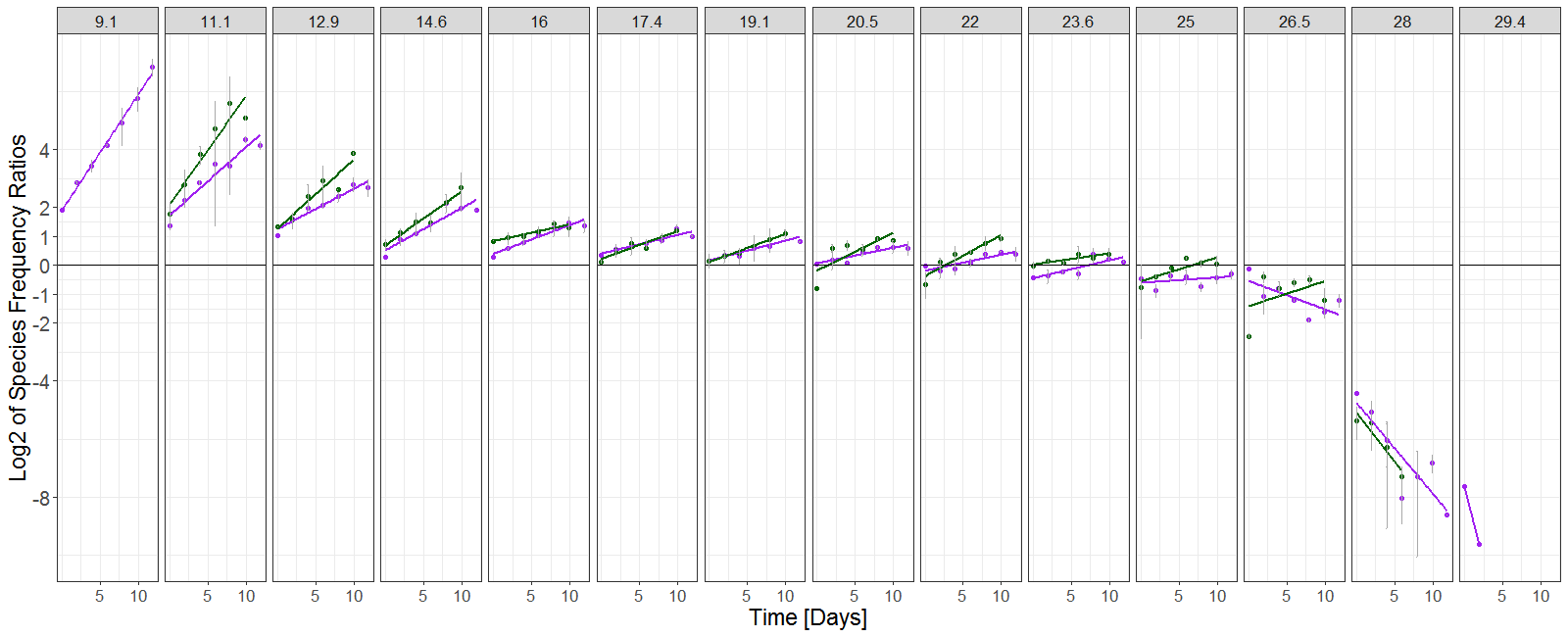


Supplementary Figure S11: Log_2_ over time of the *P. tricornutum*-to-*T. pseudonana* cell abundance ratios in low N mixed cultures. Purple lines are the first 2 competition replicates that were started two weeks after growing monocultures in N-limited conditions. Green lines are competition replicates that were started after the first competition was completed and monocultures had been starved for nitrate even longer.


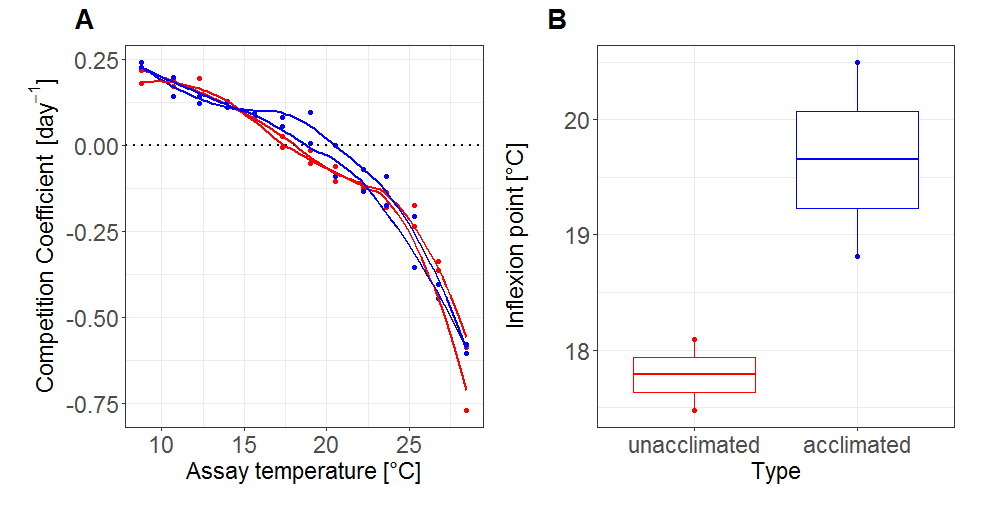


**Supplementary Figure S12:** (A) LOESS fits of high N single competition replicates across temperatures for competition coefficients calculated from observed changes in species frequency ratios (Suppl. Fig. 10). (B) Average temperatures (n=2) of unacclimated and acclimated inflexion points of competitive advantage. The ANOVA output test for a significant difference between the two groups can be found in Supplementary Table S5.
